# Hydrogen sulphide alleviates iron deficiency by promoting iron availability and plant hormone levels in *Glycine max* seedlings

**DOI:** 10.1186/s12870-020-02601-2

**Published:** 2020-08-20

**Authors:** Juan Chen, Ni-Na Zhang, Qing Pan, Xue-Yuan Lin, Zhouping Shangguan, Jian-Hua Zhang, Ge-Hong Wei

**Affiliations:** 1 State Key Laboratory of Soil Erosion and Dryland Farming on the Loess Plateau, Northwest A&F University, Yangling, Shaanxi 712100 P.R. China; 2 School of Life Sciences and State Key Laboratory of Agrobiotechnology, the Chinese University of Hong Kong, Shatin, Hong Kong; 3 Department of Biology, Hong Kong Baptist University, Kowloon Tong, Hong Kong

**Keywords:** Hydrogen sulphide (H_2_S), Iron assimilation, Iron deficiency, Organic acid, Plant hormones, Soybean (*Glycine max*)

## Abstract

**Background:**

Hydrogen sulphide (H_2_S) is involved in regulating physiological processes in plants. We investigated how H_2_S ameliorates iron (Fe) deficiency in soybean (*Glycine max* L.) seedlings. Multidisciplinary approaches including physiological, biochemical and molecular, and transcriptome methods were used to investigate the H_2_S role in regulating Fe availability in soybean seedlings.

**Results:**

Our results showed that H_2_S completely prevented leaf interveinal chlorosis and caused an increase in soybean seedling biomass under Fe deficiency conditions. Moreover, H_2_S decreased the amount of root-bound apoplastic Fe and increased the Fe content in leaves and roots by regulating the ferric-chelate reductase (FCR) activities and Fe homeostasis- and sulphur metabolism-related gene expression levels, thereby promoting photosynthesis in soybean seedlings. In addition, H_2_S changed the plant hormone concentrations by modulating plant hormone-related gene expression abundances in soybean seedlings grown in Fe-deficient solution. Furthermore, organic acid biosynthesis and related genes expression also played a vital role in modulating the H_2_S-mediated alleviation of Fe deficiency in soybean seedlings.

**Conclusion:**

Our results indicated that Fe deficiency was alleviated by H_2_S through enhancement of Fe acquisition and assimilation, thereby regulating plant hormones and organic acid synthesis in plants.

## Background

Iron (Fe) is an essential micronutrient for both the growth and development of plants and the health life of human beings. Fe, as a universal cofactor, plays an vital role in photosynthesis, respiration, hormone biosynthesis, and morphogenesis cellular enzymatic reactions and the growth and developmental processes of plants [1, 2]. Although Fe is rich in soil, it is often difficult for crops to uptake and utilize because of the low solubility of oxidized Fe(III) in compound form in alkaline soil [3]. Additionally, Fe deficiency causes the interveinal chlorosis of leaf and the impairment of chlorophyll biosynthesis, and further influences photosynthesis and the growth and development in plants [4]. Therefore, the uptake and availability of Fe is closely related to the plant growth and crop productivity.

Fe absorption, transport and circulation can strictly control Fe abundance in plant cells [5, 6]. Plants have evolved two distinct mechanisms for obtaining Fe from the rhizosphere to adapt to Fe-deficient environments. Strategy I plant species (all dicots and non-graminaceous monocots) respond to Fe deficiency in at least three steps: (1) the release of protons to acidify the rhizosphere by H^+^-ATPase, (2) the induction of ferric chelate reductase activity mediated by ferric-chelate reductase (FCR), which catalyses the reduction of ferric iron chelates to Fe^2+^, and (3) the uptake of Fe^2+^ by the high-affinity metal transporter IRT1 (iron-regulated transporter 1), which is responsible for transporting the Fe^2+^ into root cells [3, 7, 8]. Strategy II plant species (graminaceous monocots) respond to Fe deficiency in four steps: (1) phytosiderophore (muginetic acid, MAs) biosynthesis within roots, (2) phytosiderophore (PS) secretion into the rhizosphere, (3) solubilization of insoluble Fe in soils through the chelation of PSs, and (4) uptake of the ferric-phytosiderophore complex by roots [9, 10].

Plants have evolved a series of physiological and morphological responses to maintain the Fe dynamic balance in Fe-deficient soils. For example, the basic helix-loop-helix (bHLH), as a transcriptional regulatory element, has been identified to regulate the adaption of Fe deficiency in plants [11]. Fe deficiency can rapidly induce high expression levels of *bHLHs*, while subsequent Fe re-supplementation can inhibit *bHLH* expression [12]. Additionally, ferritin is an iron storage protein that is localized in plastids and plays roles during development and under stress conditions. Moreover, ferritin Fe in leaves may serve as a preliminary pool for building up of Fe-containing proteins, and ferritin accumulation maintains Fe homeostasis and protects against iron-mediated oxidative stress and abiotic stress [11, 13]. In *Arabidopsis thaliana*, ferritins are encoded by a multigene family [14]. Among the ferritins, the expression level of *AtFer1* is increased in response to Fe application [15].

Several plant hormones are particularly involved in the process of Fe deficiency responses [11, 16]. For instance, auxin plays vital roles in the morphological changes in the root system in response to Fe deficiency, and Fe deficiency causes an increase in the auxin synthesis [17]. Fe deficiency significantly induces the over-production of ethylene, and the transcriptional regulation of a series of Fe acquisition genes is modulated by ethylene [18, 19]. Abscisic acid (ABA) alleviates Fe deficiency-induced chlorosis by promoting Fe transport and redistribution from roots to shoots [20]. In addition, three other hormones, including cytokinin (CTK), jasmonate (JA), and brassinosteroids (BRs), can negatively regulate Fe deficiency responses in plants [21, 22]. For instance, both exogenous CTK and methyl-JA treatments obviously inhibit the expression levels of Fe acquisition-related genes in plants, such as *IRT*, *FRO2*, and *FIT* [21, 23]. Similarly, BRs, as steroid hormones, play an important role in the plant response to various environmental stresses. Wang et al. [24] found that a BR biosynthesis-defective mutant displays a greater tolerance to Fe deficiency than wild-type plants. Shen et al. [25] reported that SA activates Fe translocation to adapt to the Fe-deficient environment. However, whether other signalling molecule interactions with plant hormones are involved in regulating Fe assimilation is still not clear.

Recently, many studies have shown that hydrogen sulphide (H_2_S), as a signalling molecule similar to nitric oxide (NO) and carbon monoxide (CO), plays an important role in various biological processes in plants. For instance, previous studies have revealed that H_2_S promotes seed germination [26], alleviates oxidative damage against copper and aluminium stress [27, 28], counteracts chlorophyll loss [29], and alleviates osmotic stress by enhancing antioxidant enzyme stress in sweet potato seedling leaves [30]. Furthermore, salinity toxicity is alleviated by H_2_S in plants [31, 32]. H_2_S induces drought tolerance by enhancing polyamines and sugar biosynthesis in *Spinacia oleracea* seedlings [33]. In addition, zinc (Zn) toxicity, cadmium (Cd) toxicity, and heat stress are alleviated by H_2_S [34–37]. H_2_S induces stomatal closure and participates in abscisic acid (ABA)-dependent signalling pathway in guard cells [38]. Our previous study has shown that H_2_S enhances photosynthesis in *Spinacia oleracea* seedlings [39].

Previously published studies have revealed that NO and CO regulates Fe metabolism by affecting FCR activities and Fe assimilation-related gene expression in plants [11, 18, 40]. Interestingly, H_2_S improves strategy II plant (*Zea mays*) adaptation to Fe deficiency by increasing Fe uptake in roots and transport in leaves [10]. However, in contrast to strategy II plant, whether H_2_S is involved in regulating strategy I plant responses to Fe deficiency is still unclear.

The aim of the present work was to investigate how H_2_S ameliorate Fe deficiency in soybean seedlings. Our hypothesis was that H_2_S has a beneficial influence on soybean seedlings in response to Fe deficiency. To test this hypothesis, we investigated the effect of H_2_S on Fe storage and availability in the roots of soybean seedlings using physiological and transcriptomics methods.

## Results

### NaHS inhibits dry biomass and chlorophyll loss in iron-deficient *Glycine max* plants

**Fig. 1:**
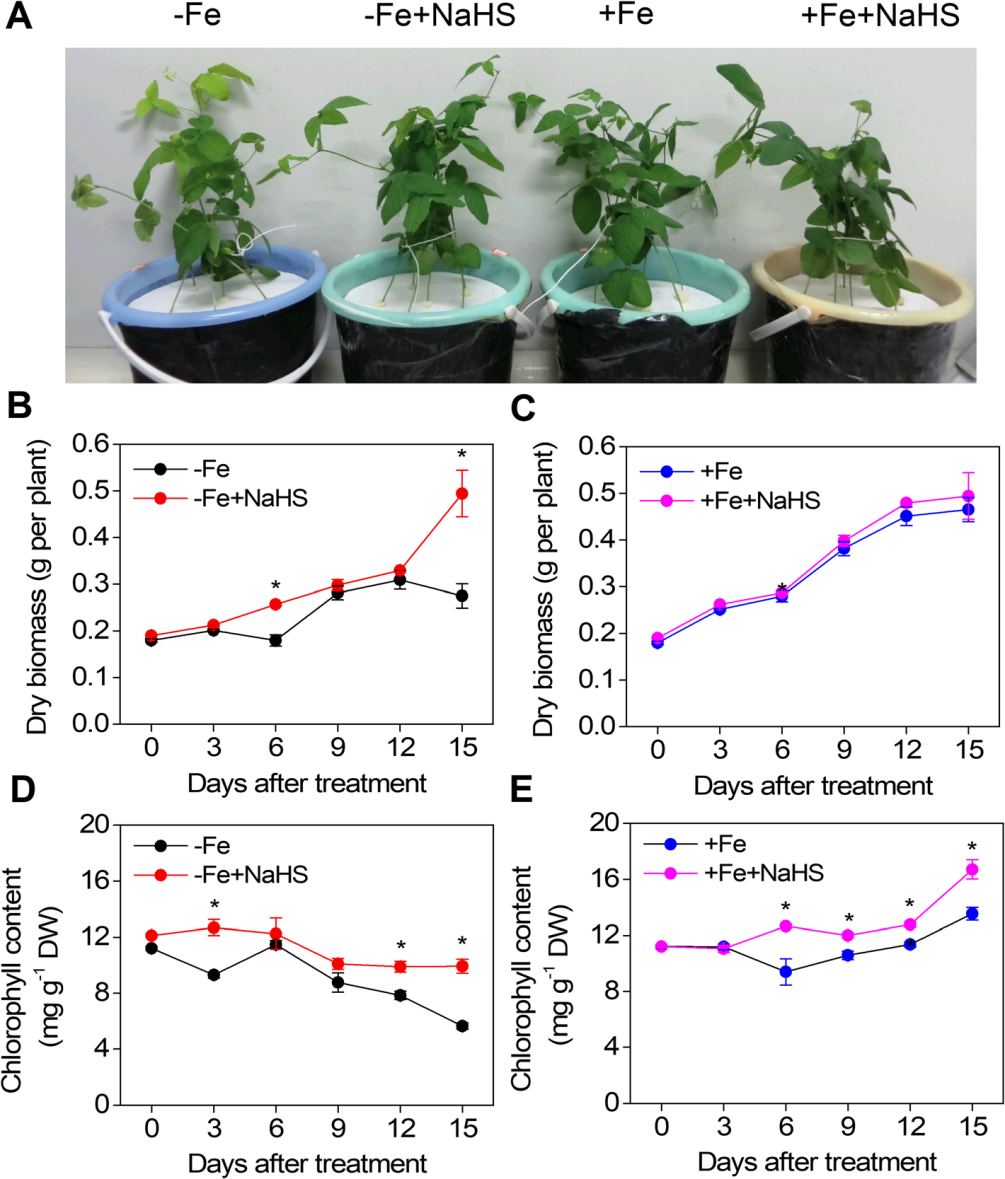
Effect of NaHS on the phenotype (**a**), dry biomass (**b** and **c**), and chlorophyll content (**d** and **e**) of iron-deficient *Glycine max* plants. The 3-w-old *G. max* seedlings that were treated with the nutrition solution containing 1 μM Fe(III)-EDTA (−Fe) or 50 μM Fe(III)-EDTA (+Fe) with or without 100 μM NaHS for 15 d. B and C show the dry weight of the –Fe or + Fe plants, respectively. D and E show the chlorophyll content of the –Fe or + Fe plants, respectively. Error bars represents the mean ± SE. * indicates a significant difference at *P* < 0.05. –Fe, 1 μM Fe; −Fe + NaHS, seedlings treated with 100 μM NaHS and 1 μM Fe; +Fe, 50 μM Fe; +Fe + NaHS, seedlings treated with 100 μM NaHS and 50 μM Fe

NaHS was used as the exogenous H_2_S donor as described by Hosoki et al. [41]. NaHS successfully suppressed the symptoms of Fe deficiency in soybean plants treated with the NaHS -free nutrient solution and 1 μΜ Fe(III)-EDTA for 15 d (Fig. 1a). By contrast, plants grown without NaHS became severely chlorotic. However, under Fe-sufficient (+Fe) conditions, the effect of NaHS on the phenotype of soybean growth was not significant (Fig. 1a). After 15 d of treatments, the dry weight of leaf, stem and root were strongly enhanced by NaHS in Fe-deficient (−Fe) plants compared with those that had not been treated with NaHS, in contrast to the –Fe plants, where H_2_S did not affect the dry weight of +Fe plants (Table 1). Moreover, the chlorophyll content significantly decreased after 15 d of treatment, but NaHS significantly inhibited the Fe deficiency-induced chlorophyll loss in soybean leaves (Table 1). Moreover, under +Fe conditions, NaHS also significantly promoted the chlorophyll content of soybean leaves (Table 1). To distinguish the role of H_2_S from that of other sulphur-containing derivatives and sodium, a series of sulphur- and sodium-containing chemicals including NaHS, Na_2_S, Na_2_SO_4_, Na_2_SO_3_, NaHSO_4_, NaHSO_3_, and NaAC (100 μM) were used to treat soybean seedlings under iron deficiency conditions. After Fe deficiency treatment for 15 d, the chlorophyll content did not cause a great increase in the Na^+^ or other sulphur-containing compounds treatments compared with the NaHS treatment (Fig. S1).

**Table 1:** Effect of NaHS on chlorophyll content, dry biomass, and Fe concentration of iron-deficient *Glycine max* plants. The 3-w-old *Glycine max* seedlings were treated with the nutrition solution containing 1 μM Fe(III)-EDTA or 50 μM Fe(III)-EDTA with or without 100 μM NaHS for 15 days. Error bars represents the mean ± SE. Different letters indicate significant differences with *P* < 0.05. –Fe, 1 μM Fe; −Fe + NaHS, seedlings treated with 100 μM NaHS and 1 μM Fe; +Fe, 50 μM Fe; +Fe + NaHS, seedlings treated with 100 μM NaHS and 50 μM Fe

Treatments	Chlorophyll content	Dry biomass (g per plant)			Fe concentration (mg g^− 1^ DW)		
	(mg g-1 FW)	Leaf	Stem	Root	Leaf	Stem	Root
- Fe	0.564 ± 0.022 d	0.106 ± 0.004 b	0.118 ± 0.008 b	0.046 ± 0.001 b	0.377 ± 0.005 c	0.202 ± 0.005 c	0.309 ± 0.013 d
- Fe + NaHS	0.992 ± 0.051 c	0.186 ± 0.007 a	0.209 ± 0.002 a	0.080 ± 0.002 a	0.490 ± 0.028 b	0.244 ± 0.021 b	0.399 ± 0.022 c
+ Fe	1.353 ± 0.046 b	0.174 ± 0.006 a	0.214 ± 0.024 a	0.084 ± 0.008 a	0.696 ± 0.033 a	0.425 ± 0.035 a	0.855 ± 0.113 b
+ Fe + NaHS	1.670 ± 0.068 a	0.181 ± 0.005 a	0.195 ± 0.003 a	0.083 ± 0.001 a	0.634 ± 0.041 a	0.394 ± 0.018 a	1.805 ± 0.107 a

Furthermore, to further elucidate the mechanism of the H_2_S-mediated amelioration of Fe deficiency stress time-course experiments were conducted to study the dynamics of relevant physiological indexes. For instance, under –Fe conditions, total dry biomass had an obvious increase in the NaHS-treated plants under –Fe condition for 15 d (Fig. 1b), and keep approximately the same high concentration of chlorophyll (1.2 mg g^− 1^ FW) under only +Fe treatment (Fig. 1d and e). However, under +Fe conditions, NaHS did not significantly change the dry biomass during all the treatment period, but the chlorophyll content was obviously increased by NaHS (Fig. 1c and e).

### NaHS treatment increases iron accumulation in *Glycine max* plants

The Fe concentration in the NaHS-treated plants was obviously higher than in the controls (plants not treated with NaHS) under –Fe conditions for 15 d (Table 1). For example, the Fe concentration increased by 30% in the leaves of NaHS-treated plants compared with those not treated with NaHS (Table 1). Moreover, a more than two-fold increase was found in the roots of NaHS-treated plants under +Fe conditions, but the Fe concentration in leaves and stems was not affected by NaHS (Table 1).

### NaHS modulates the pH of root bathing solution, FCR activity, and root apoplastic Fe content to iron acquisition in *Glycine max* plants

**Fig. 2:**
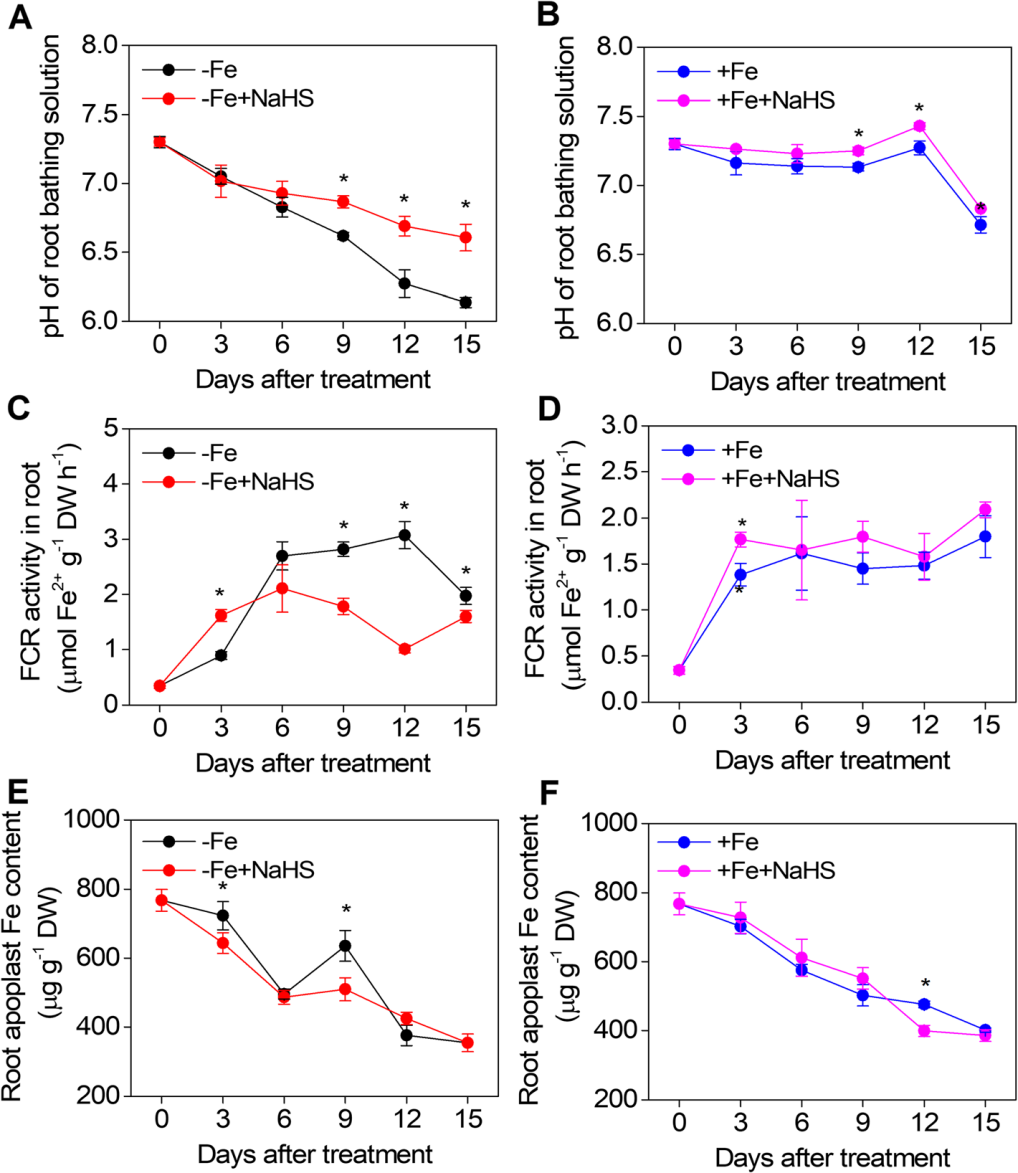
Effect of NaHS on the pH of the root bathing solution (**a** and **b**), ferric-chelate reductase (FCR) activity (**c** and **d**), and root apoplast Fe content (**e** and **f**) of iron-deficient *Glycine max* plants. The 3-w-old *G. max* seedlings were treated with the nutrition solution containing 1 μM Fe(III)-EDTA or 50 μM Fe(III)-EDTA with or without 100 μM NaHS for 15 d. A and B show the pH of the root bathing solution of the –Fe or + Fe plants, respectively. C and D show ferric-chelate reductase (FCR) activity of the –Fe or + Fe plants, respectively. E and F show the root apoplast Fe content of the –Fe or + Fe plants, respectively. Error bars represents the mean ± SE. * indicates a significant difference at *P* < 0.05. –Fe, 1 μM Fe; −Fe + NaHS, seedlings treated with 100 μM NaHS and 1 μM Fe; +Fe, 50 μM Fe; +Fe + NaHS, seedlings treated with 100 μM NaHS and 50 μM Fe

During the whole period of –Fe treatment, the pH of the root bathing solution drastically decreased from 7.3 to 6.1, but the NaHS-treated plants only slowly decreased compared with those in the –Fe treatment alone (Fig. 2a). However, under +Fe treatment, the pH of bathing solution showed significant differences between the +Fe and + Fe + NaHS treatments at both 9 and 12 d, but no significant difference was found at any other time points were (Fig. 2b). The FCR activity varied greatly depending on the NaHS treatments during the whole period of –Fe treatment (Fig. 2c). During the first 3 d, the FCR activity became significantly higher in the NaHS-treated plants than in the –Fe treatment alone (Fig. 2c). However, from the 6th d to the end of the experiment, the FCR activity gradually decreased in the NaHS-treated plants (Fig. 2c). Under +Fe treatment, NaHS did not significantly affect the FCR activity during the whole experiment period except for 3 d (Fig. 2d). Apoplastic Fe is one of the major Fe pools in plants. The amount of apoplastic Fe in the root was reduced by 11.1 and 19.8% at 3 d and 9 d in the NaHS-treated plants compared with the –Fe treatment alone, respectively (Fig. 2e). Under +Fe conditions, NaHS did not significantly affect the apoplastic Fe content in the root throughout the experimental period except at 12 d (Fig. 2f).

### Effect of NaHS on endogenous H_2_S, GSH, and NPTs content in iron-deficient *Glycine max* plants

A high accumulation of endogenous H_2_S in soybean seedlings leaves and roots caused by exogenous applied NaHS was observed under the –Fe and + Fe conditions (Table 2). Moreover, the NPT content in leaves and roots was increased by exogenous application of NaHS under the –Fe and + Fe conditions (Table 2). Additionally, reduced GSH contents in leaves and root were increased 5.3-fold and 14% by NaHS under –Fe conditions, respectively. Similarly, under +Fe conditions, the GSH content in leaves and roots was enhanced by NaHS in soybean seedlings (Table 2).

**Table 2:** Effect of NaHS on endogenous hydrogen sulfide (H_2_S), non-protein thiols (NPTs), and glutathione (GSH) content in leaves and roots of iron-deficient *Glycine max* plants. The 3-w-old *Glycine max* seedlings were treated with the nutrition solution containing 1 μM Fe(III)-EDTA or 50 μM Fe(III)-EDTA with or without 100 μM NaHS for 15 days. Error bars represents the mean ± SE. Different letters indicate significant differences with *P* < 0.05. –Fe, 1 μM Fe; −Fe + NaHS, seedlings treated with 100 μM NaHS and 1 μM Fe; +Fe, 50 μM Fe; +Fe + NaHS, seedlings treated with 100 μM NaHS and 50 μM Fe

Treatment	Endogenous H_2_S content (μmol g^− 1^ FW)		NPTs (μmol g^− 1^ FW)		GSH (nmol g^− 1^ FW)	
	Leaf	Root	Leaf	Root	Leaf	Root
-Fe	0.046 ± 0.013 b	0.013 ± 0.004 C	7.56 ± 0.20 b	3.74 ± 0.10 B	28.56 ± 5.71 c	164.4 ± 9.71 B
-Fe + NaHS	0.123 ± 0.014 a	0.075 ± 0.010 A	8.95 ± 0.29 a	4.88 ± 0.44 A	149.5 ± 15.6 b	187.1 ± 6.62 A
+Fe	0.056 ± 0.007 b	0.029 ± 0.002 B	7.96 ± 0.64 ab	4.05 ± 0.27 B	162.3 ± 10.7 b	166.2 ± 10.8 B
+Fe + NaHS	0.119 ± 0.010 a	0.098 ± 0.017 A	8.37 ± 0.25 a	5.28 ± 0.26 A	240.2 ± 15.7 a	182.4 ± 5.34 A

### Transcriptome analysis of the effect of NaHS on the expression of different genes in *Glycine max* plants in response to iron deficiency

We constructed RNA-Seq libraries for 12 samples (3 replicates per treatment). These RNA libraries were sequenced using an Illumina Hiseq 4000 platform, and a total of 50,315,998 (−Fe), 56,902,806 (−Fe + NaHS), 60,885,868 (+Fe), and 50,140,356 (+Fe + NaHS) sequence reads were generated (Table S1). The replication of the data in every treatment was quite high, and the R^2^ was approximately 0.9 (Fig. S6). An overview of the sequencing and assembly was outlined in Table S1 and Table S2. We pooled the short reads and aligned them against the soybean genome and found that approximately 87.31–89.19% of the reads mapped to genes (Table S1).

**Fig. 3:**
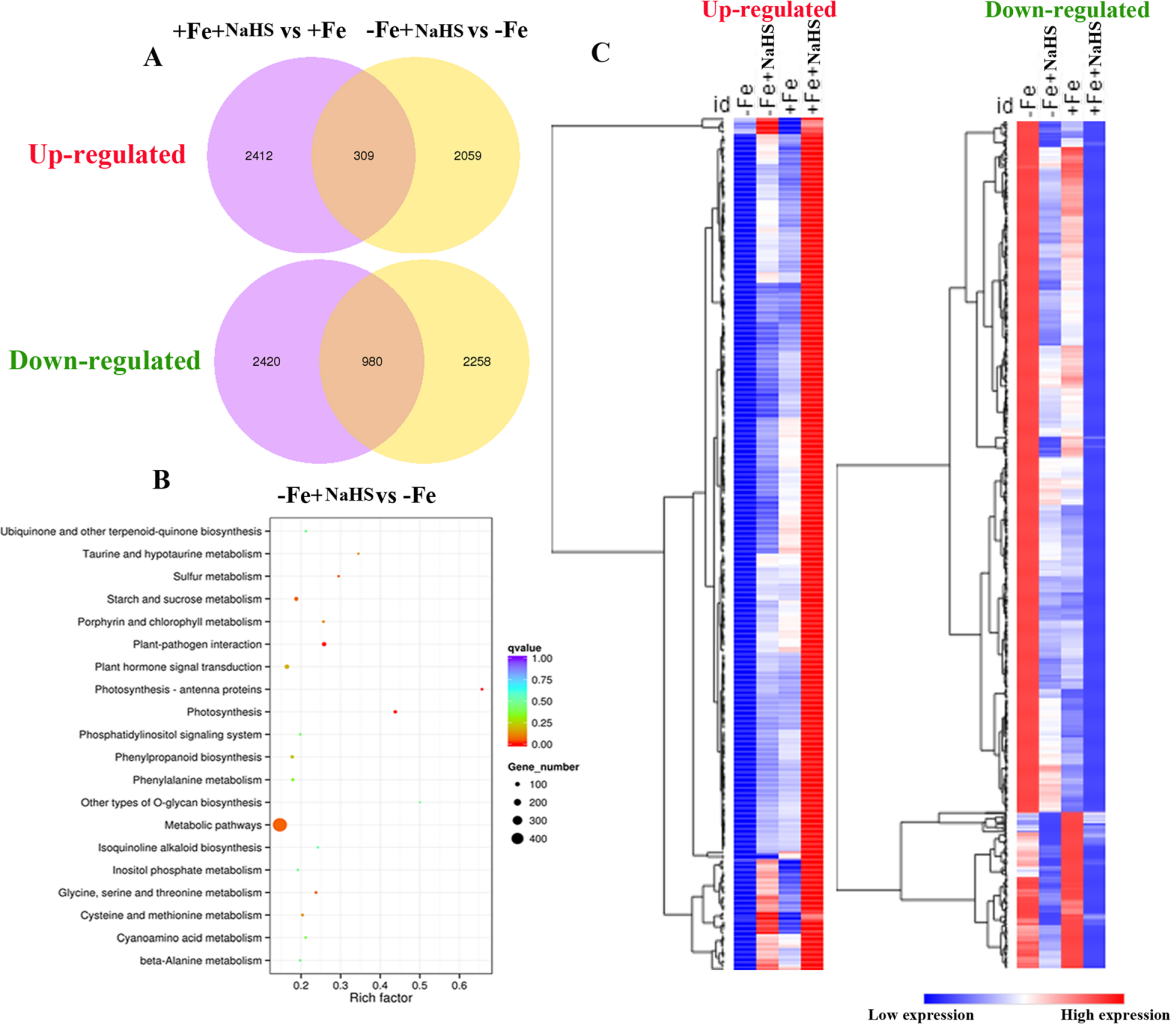
The global transcriptome responds specifically to iron deficiency and NaHS treatments. **a** Venn diagrams showing the DEGs that were up-regulated and down-regulated by NaHS treatment under iron deficiency and iron sufficiency conditions in roots of *Glycine max* plants. **b** Specific significantly enriched Kyoto Encyclopedia of Genes and Genomics (KEGG) pathways for DEGs by NaHS treatment under iron deficiency condition (**c**) Hierarchical clustering of the up-regulated of 309 DEGs (left) and down-regulated of 980 DEGs (right) by NaHS treatment under iron deficiency and iron sufficiency conditions in roots of *Glycine max* plants. –Fe, 1 μM Fe; −Fe + NaHS, seedlings treated with 100 μM NaHS and 1 μM Fe; +Fe, 50 μM Fe; +Fe + NaHS, seedlings treated with 100 μM NaHS and 50 μM Fe. More information can be found in the supplementary material

Based on the deep sequencing of the 12 libraries in the present study, 54,718 genes were detected from soybean roots in the Fe deficiency treatment, which covered from at least 82% of the reference genes in soybean. Moreover, under –Fe conditions, the application of NaHS affected 5606 DEGs, of which 2368 were up-regulated genes and 3238 were down-regulated genes (Fig. S7A). Similarly, under +Fe conditions, NaHS regulated 6121 DEGs, of which 2721 were up-regulated genes and 3400 were down-regulated genes (Fig. S7B). Among these DEGs, 309 and 980 DEGs in soybean roots were co-upregulated and -downregulated by NaHS under –Fe and + Fe conditions, respectively (Fig. 5a). These up-regulated and down-regulated DEGs are clustered in Fig. 3c. The detailed information is listed in Table S5–8. Additionally, to understand the function of DEGs, we used a KEGG pathway network to identify their enrichment among different metabolic pathways. Significantly enriched KEGG pathways were identified using a *P*-value based on the hypergeometric distribution. For instance, under –Fe conditions, twenty pathways were significantly enriched by NaHS treatment. This KEGG pathways network analysis showed that “sulphur metabolism”, “plant hormone signal transduction”, “metabolism”, “porphyrin and chlorophyll metabolism”, and “cysteine and methionine”, among others, were significantly enriched under –Fe conditions (Fig. 3b). Meanwhile, the GO analysis had listed in Fig. S8.

### NaHS regulates the expression of iron homeostasis-related genes in iron-deficient *Glycine max* plants

**Fig. 4:**
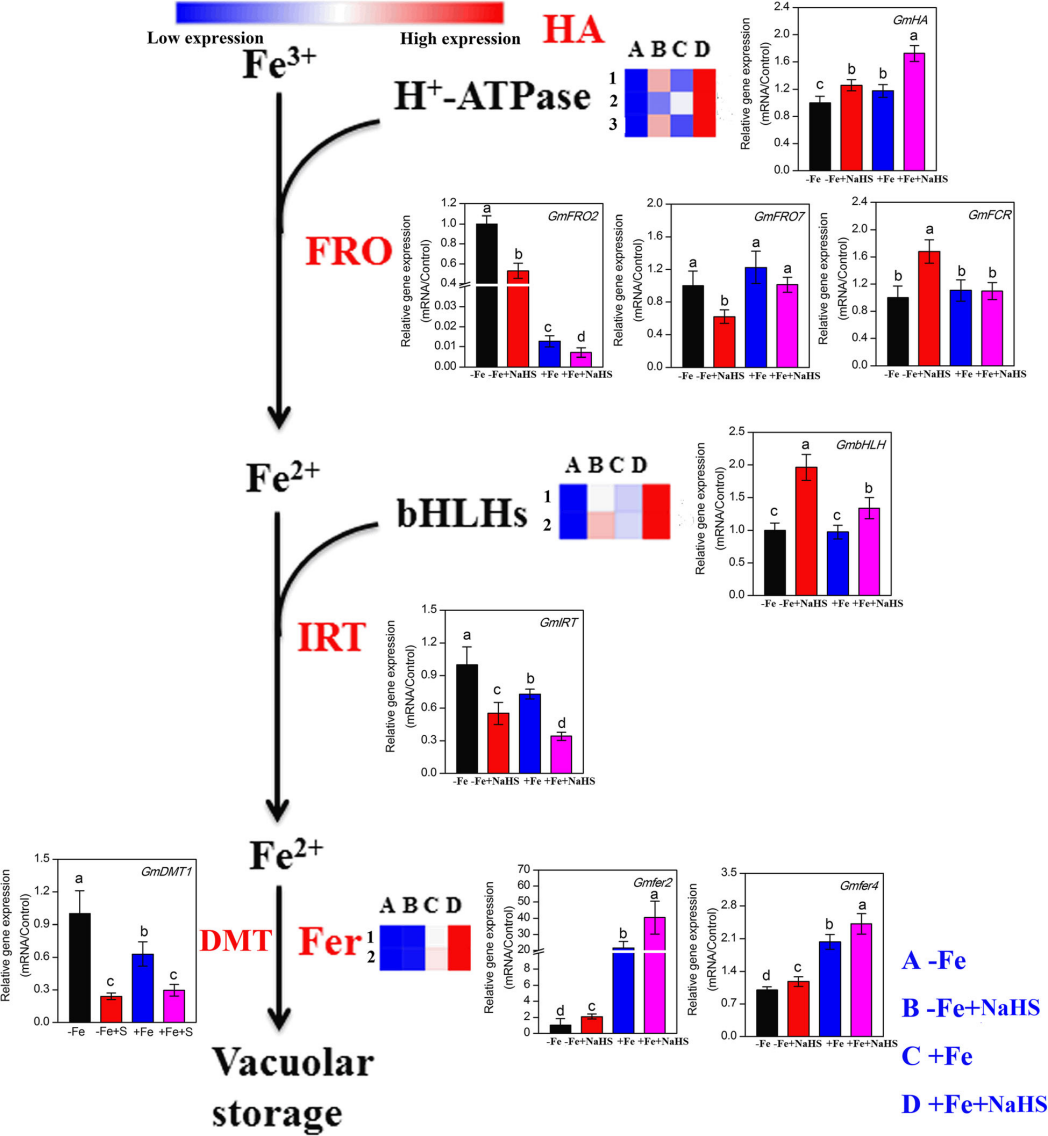
Genes in the iron assimilation pathway were regulated by NaHS treatment under iron deficiency and iron sufficiency conditions in roots of *Glycine max* plants. Transcript levels of genes are listed in the figure, and the detailed DEGs are listed in supplementary Table S5. The data of columns indicated the gene expression of iron assimilation using RT-PCR. The relative mRNA levels of each gene were normalized to the mRNA of *GmEIF1B and Gmactin*. Data are presented as the mean ± SE (*n* = 3). Columns labelled with different letters indicate significant differences at *P* < 0.05. Abbreviations of gene names are provided in supplementary Table S3. A: –Fe, 1 μM Fe; B: –Fe + NaHS, seedlings treated with 100 μM NaHS and 1 μM Fe; C: +Fe, 50 μM Fe; D: +Fe + NaHS, seedlings treated with 100 μM NaHS and 50 μM Fe

The transcriptome data showed that iron homeostasis-related genes, including 3 *H*^*+*^*-ATPase* (*HA*) genes, 2 *bHLHs* genes, and 2 ferritins genes (*fer*), were regulated by NaHS under –Fe and + Fe conditions (the transcriptome data are presented using clustering in Fig. 4). To further confirm the transcriptome result, the qRT-PCR method was used (the qRT-PCR data are presented using column graphs in Fig. 4). As shown in Fig. 4, the expression levels of 3 *GmHAs* genes were up-regulated in the NaHS treatment under –Fe and + Fe conditions. Moreover, the qRT-PCR results also showed that NaHS significantly promoted the transcript abundances of the *GmHA* gene under the same conditions. Additionally, the expression abundances of *GmFRO2* and *GmFRO7* were obviously decreased by NaHS under –Fe conditions. By contrast, the expression levels of the *GmFCR* were significantly enhanced by NaHS under the same conditions. Moreover, under +Fe conditions, NaHS affected the expression abundances of *GmFRO7* and *GmFCR* in the roots of soybean seedlings. In addition, the transcriptome data showed that the expression levels of 2 *GmbHLHs* were obviously increased by NaHS under –Fe and + Fe conditions, and the qRT-PCR analysis further confirmed these results. The expression levels of *GmIRT* and *GmDMT1* were significantly down-regulated by NaHS under –Fe and + Fe conditions. Finally, the 2 *Gmfers* expression levels were not affected by NaHS under –Fe conditions, but under +Fe conditions the expression levels of these two genes were significantly increased compared with the –Fe treatment. Similarly, the qRT-PCR analysis also indicated that Fe and NaHS could obviously increase the expression levels of *Gmfer2* and *Gmfer4* genes in the roots of soybean seedlings.

### NaHS affects the expression of Sulphur metabolism-related genes in iron-deficient *Glycine max* plants

**Fig. 5:**
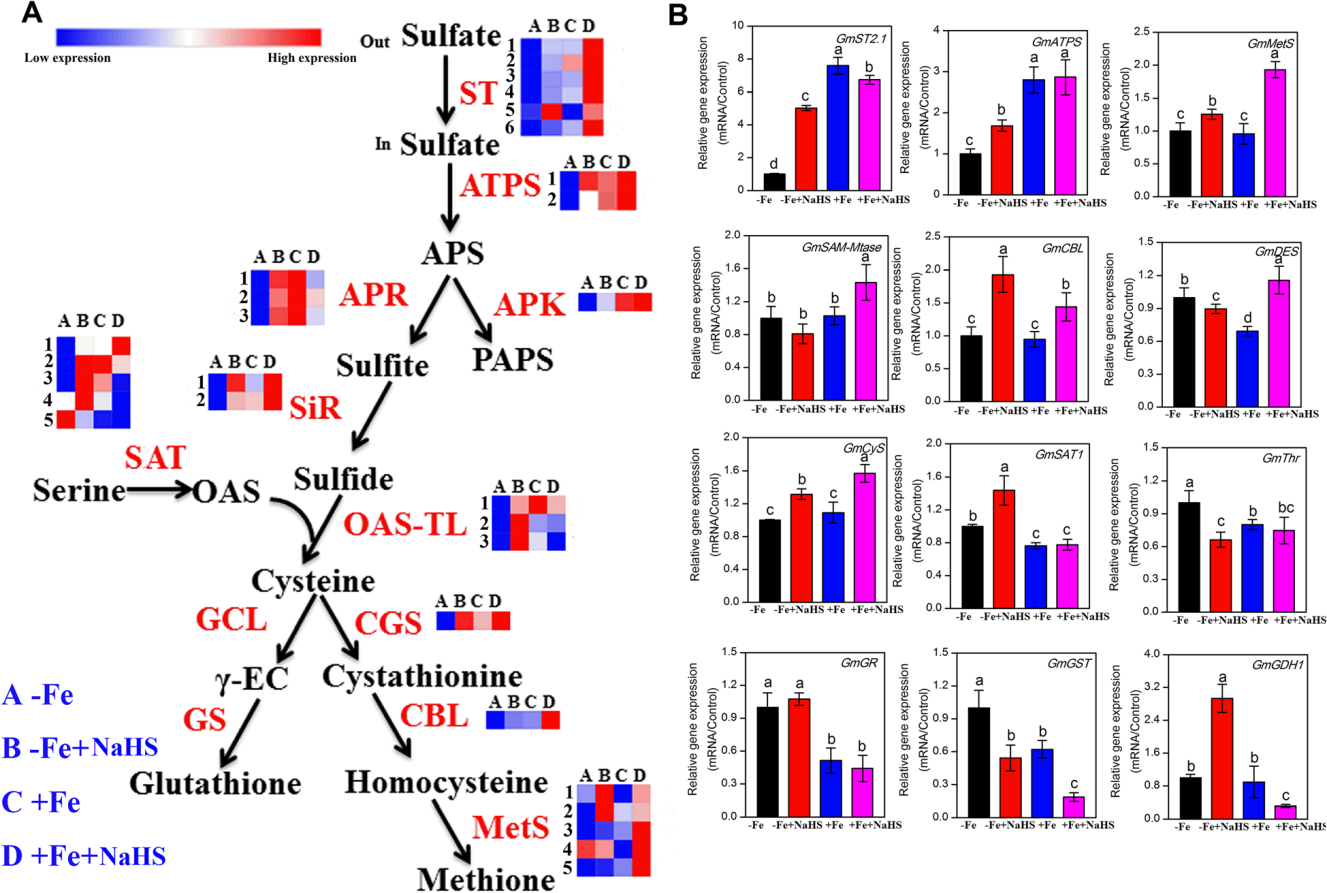
Genes in the sulfur assimilation pathway were regulated by NaHS treatment under iron deficiency and iron sufficiency conditions in roots of *Glycine max* plants. **a** Transcript levels of genes were listed in this figure and the detailed DEGs are listed in supplementary Table S6. **b** The gene expression of sulphur assimilation in roots of *Glycine max* plants using RT-PCR under iron deficiency conditions. The relative mRNA levels of each gene were normalized to the mRNA of *GmEIF1B and Gmactin*. Data are presented as the mean ± SE (n = 3). Columns labelled with different letters indicate significant differences at *P* < 0.05. Abbreviations of gene names are provided in supplementary Table S3. A: –Fe, 1 μM Fe; B: –Fe + NaHS, seedlings treated with 100 μM NaHS and 1 μM Fe; C: +Fe, 50 μM Fe; D: +Fe + NaHS, seedlings treated with 100 μM NaHS and 50 μM Fe

Sulphur (S) metabolism-related genes, including *GmST*, *GmATPS*, *GmAPK*, *GmAPR*, *GmSiR*, *GmSAT*, *GmOAS-TL*, *GmCGS*, *GmCBL*, and *GmMetS*, are modulated by NaHS in the roots of soybean under –Fe and + Fe conditions using the transcriptome method (Fig. 5a). The expression abundances of 6 *GmSTs* genes were significantly enhanced by NaHS under –Fe and + Fe conditions in the roots of soybean (Fig. 5a). Similarly, the qRT-PCR results showed that NaHS significantly up-regulated the expression of the *GmST2.1* in the roots of –Fe plants, but this gene expression level slightly decreased under +Fe conditions (Fig. 5b). The expression levels of 2 *GmATPS* genes showed obvious increases in the NaHS-treated plants compared with the plants that were not treated with NaHS in the presence or absence of Fe in the roots of soybean seedlings; the qRT-PCR analysis further confirmed these results (Fig. 5). Additionally, NaHS obviously increased the expression levels of 3 *GmAPRs* genes in the roots of –Fe plants. However, under +Fe conditions, the expression levels of these three genes were obviously decreased by NaHS. Similarly, the expression of the 2 *GmSiR* and *GmAPK* genes were up-regulated by NaHS under –Fe and + Fe conditions. The expression levels of five *GmSAT* genes were changed by NaHS in the roots of –Fe and + Fe plants, and the qRT-PCR data indicated that the *GmSAT1* gene expression level was up-regulated by NaHS under –Fe conditions, but was not affected in the +Fe plants (Fig. 5). Moreover, the expression abundances of 3 *GmOAS-TL* genes were enhanced by the NaHS treatment in the –Fe plants, but these three genes were significantly down-regulated under +Fe conditions. The gene expression levels of *GmCyS* were up-regulated by NaHS under –Fe and + Fe conditions. Moreover, the expression abundances of *GmCGS* and *GmCBL* were obviously induced by NaHS in the roots of soybean seedlings, and the qRT-PCR data further confirmed this result. Interestingly, NaHS treatment slightly inhibited the expression level of the *GmDES* gene under –Fe conditions, but this gene expression level was significantly up-regulated by NaHS under +Fe conditions. Finally, the expression abundances of 5 *GmMetSs* genes were affected to different degrees by NaHS under –Fe and + Fe conditions, and the qRT-PCR data indicated that NaHS treatment could up-regulate their gene expression abundances in the roots of soybean seedlings. In addition, we also analyzed other thio- or stress-related genes, including *GmSAM-Mtase*, *GmThr, GmGR*, *GmGST*, and *GmGDH1*, in the roots of –Fe plants using qRT-PCR. The expression levels of *GmThr* and *GmGST* were significantly down-regulated by NaHS under –Fe conditions in the roots of soybean seedlings, but under the same conditions NaHS treatment did affect the expression abundances of the *GmSAM-Mtase* and *GmGR* genes. However, the expression level of the *GmGDH1* gene was significantly up-regulated by NaHS under –Fe conditions, while the opposite result was observed under +Fe conditions.

### Plant hormones play an important role in NaHS-induced adaption to iron deficiency in *Glycine max* plants

**Fig. 6:**
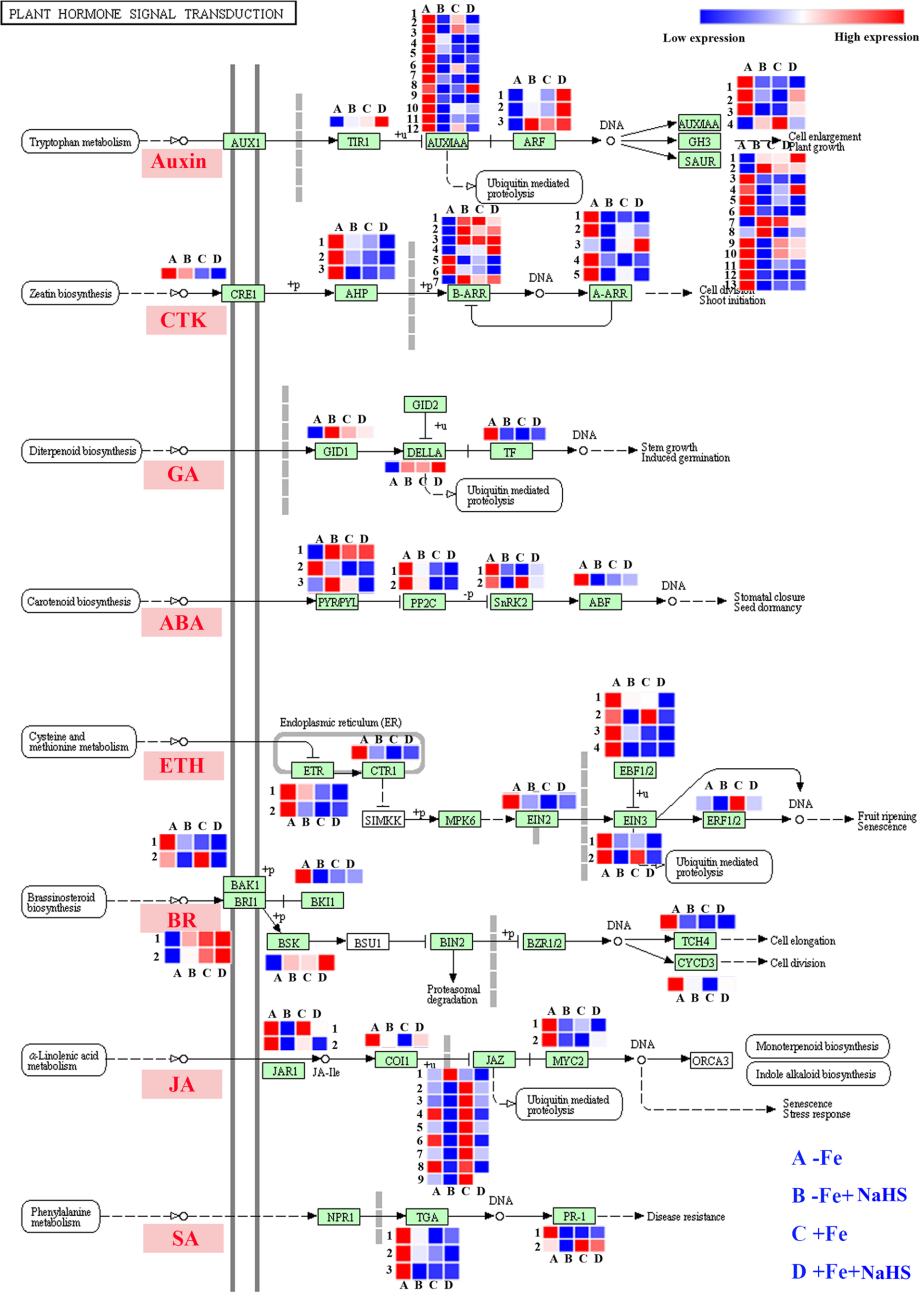
Genes in the plant hormones pathway were regulated by NaHS treatment under iron deficiency and iron sufficiency conditions in roots of *Glycine max* plants. Transcript levels of genes are listed in the figure and the detailed DEGs are listed in supplementary Table S7. CTK, cytokinin; GA, gibberellin; ABA, abscisic acid; ETH, ethylene; BR, brassinosteroid; JA, jasmonic acid; SA, salicylic acid; A: –Fe, 1 μM Fe; B: –Fe + NaHS, seedlings treated with 100 μM NaHS and 1 μM Fe; C: +Fe, 50 μM Fe; D: +Fe + NaHS, seedlings treated with 100 μM NaHS and 50 μM Fe

As shown in Fig. 6, in the auxin pathway, the expression levels of 1 *GmTIR1* and 3 *GmARFs* genes were significantly increased by NaHS in the roots of soybean seedling grown under –Fe and + Fe conditions. By contrast, the expression abundances of 12 *GmAUX/IAA*s genes were all down-regulated by NaHS under –Fe and + Fe conditions. Similarly, most of the *GmGH3* and *GmSAUR* expression levels were obviously decreased by NaHS treatment under –Fe conditions. Moreover, in the cytokinine (CTK) pathway, NaHS treatment significantly down-regulated the expression levels of the *GmCRE1, GmAHP*, *GmA-ARR*, and 2 *GmB-ARR* genes under –Fe conditions, but the expression levels of 5 other *GmB-ARR* genes were up-regulated in the roots of soybean seedlings. By contrast, in the gibberellin (GA) pathway, the expression of *GmGID1* and *GmDELLA* were significantly up-regulated by NaHS treatment under –Fe conditions, but *GmTF* was obviously down-regulated under the same conditions. Moreover, NaHS treatment significantly decreased the gene expression levels of *GmPP2C*, *GmSnRK2*, and *GmABF* in the abscisic acid (ABA) pathway under –Fe conditions. Nevertheless, the 2 *GmPYT/PYL* genes were up-regulated in the roots of soybean seedlings. In the ethylene (ETH) pathway, the expression levels of all genes, including *GmETR*, *GmCTR1*, *GmEIN2*, *GmEBF1/2*, *GmEIN3*, and *GmERF1/2*, were significantly down-regulated by NaHS in the roots of soybean seedlings under –Fe conditions. Additionally, in the brassinosteroid (BR) pathway, the expression levels of the *GmBAK1*, *GmBKI1*, *GmTCH4*, and *GmCYCD3* genes were obviously decreased by NaHS in the roots of –Fe plants. However, the expression abundances of *GmBRI1* and *GmBSK* were enhanced under the same conditions. Moreover, under –Fe conditions, NaHS treatment significantly down-regulated all genes involved in the jasmonic acid (JA) pathway in the roots of soybean seedlings, which contains the genes *GmJAR1*, *GmCOI1*, *GmJAZ*, and *GmMYC2*. Finally, the expression abundances of *GmTGA* and *GmPR-1* were significantly down-regulated by NaHS treatment under –Fe conditions.

**Fig. 7:**
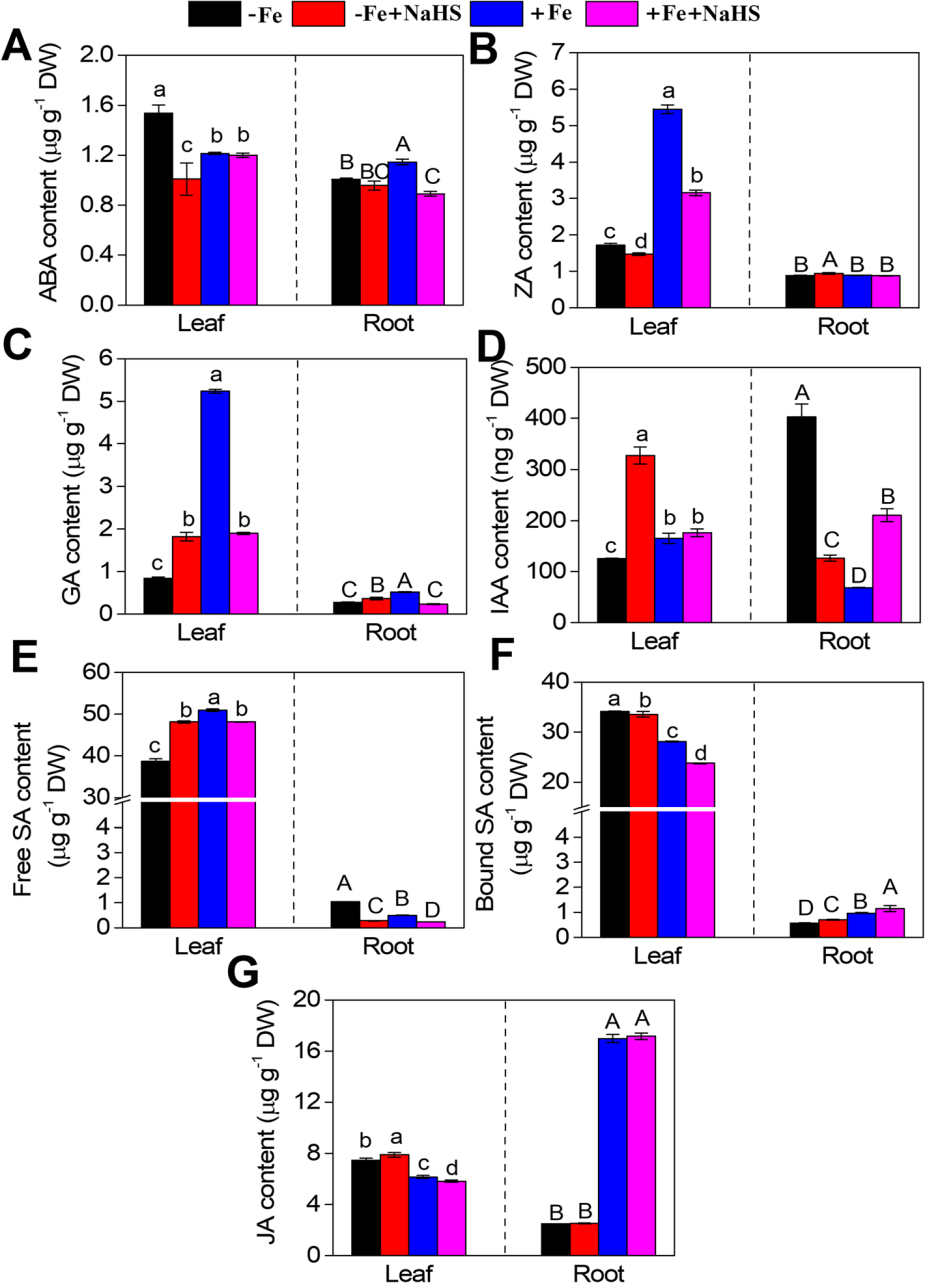
Effect of Fe deficiency and NaHS treatment on the contents of abscisic acid (ABA) (**a**), zeatin (ZA) (**b**), gibberellin (GA) (**c**), indoleacetic acid (IAA) (**d**), free salicylic acid (SA) (**e**), bound salicylic acid (SA) (**f**), and jasmonic acid (JA) (**g**) in leaves and roots of *Glycine max* plants. The 3-w-old *G. max* seedlings were treated with the nutrition solution containing 1 μM Fe(III)-EDTA or 50 μM Fe(III)-EDTA with or without 100 μM NaHS for 15 d. Error bars represents the mean ± SE. Columns labelled with different letters indicate significant differences at *P* < 0.05. –Fe, 1 μM Fe; −Fe + NaHS, seedlings treated with 100 μM NaHS and 1 μM Fe; +Fe, 50 μM Fe; +Fe + NaHS, seedlings treated with 100 μM NaHS and 50 μM Fe

The content of plant hormones was affected by NaHS under –Fe conditions (Fig. 7). For instance, NaHS treatment significantly decreased the ABA and ZA (zeatin, ZA, a natural cytokinins in plants) contents in the leaves of –Fe plants, but this difference was not obvious in the roots (Fig. 7a, b). In contrast, the GA contents in the leaves and roots of soybean seedlings were increased by NaHS under –Fe conditions, but the GA content showed an obvious decrease under +Fe conditions (Fig. 7c). Moreover, the IAA content in leaves of soybean seedlings was obviously increased by NaHS under –Fe conditions, but NaHS treatment decreased the IAA content in the roots under the same conditions (Fig. 7d). NaHS treatment promoted the free SA synthesis in the leaves but decreased the free SA content in the roots under –Fe conditions (Fig. 7e). In contrast, the bound SA content in the leaves showed a slight decrease but increased in roots under –Fe and + Fe conditions (Fig. 7f). The JA content in the leaves was slightly increased by NaHS under –Fe conditions, but the JA content was decreased under +Fe conditions (Fig. 7g). In addition, NaHS did not affect the JA content in roots under –Fe and + Fe conditions (Fig. 7g).

### NaHS regulates the expression of organic acid metabolism-related genes and the organic acid contents in iron-deficient *Glycine max* plants

**Fig. 8:**
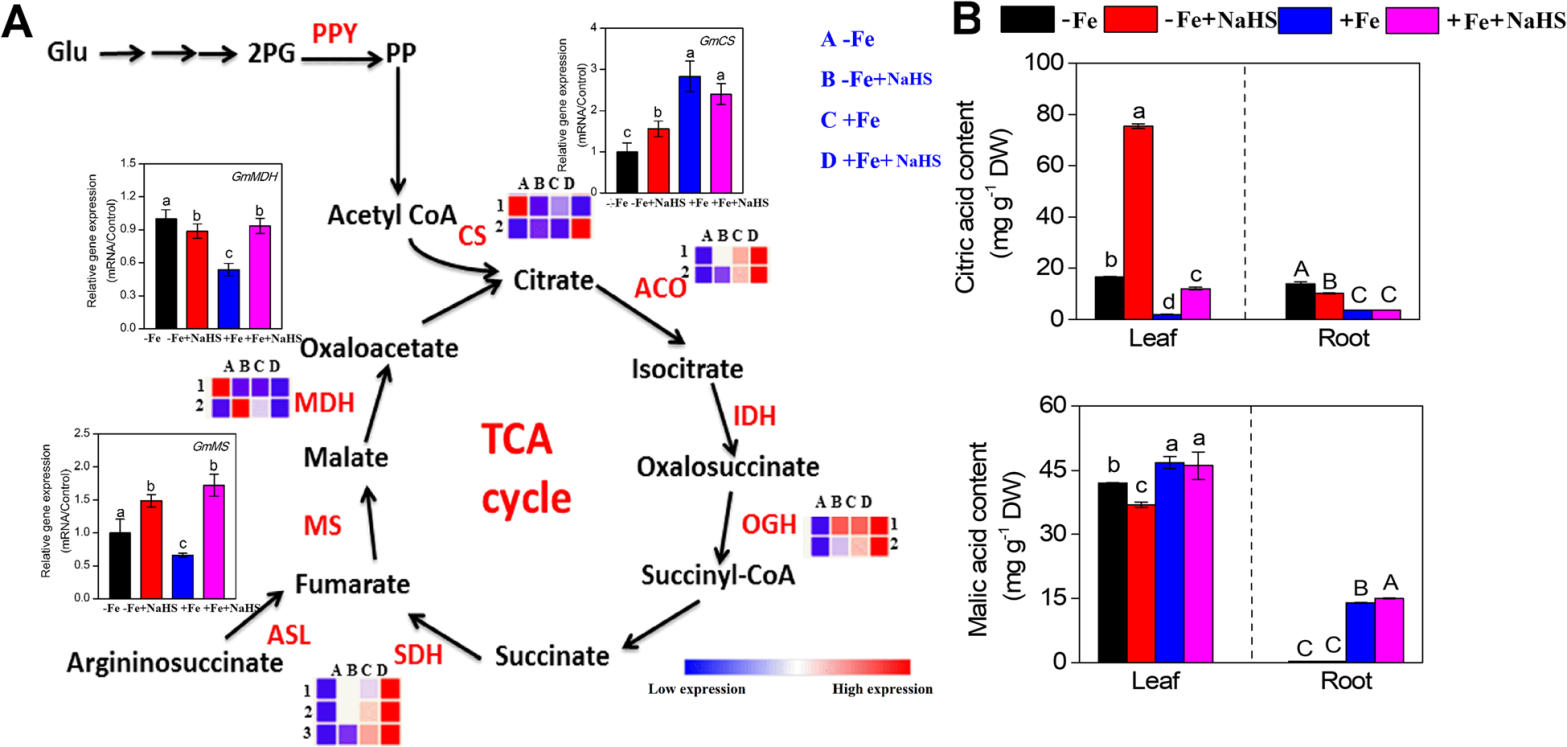
**a** Genes in the plant organic acid pathway were regulated by NaHS treatment under iron deficiency and iron sufficiency conditions in roots of *Glycine max* plants. Transcript levels of genes are listed in the figure, and the detailed DEGs are listed in supplementary Table S8. Abbreviations of gene names are provided in supplementary Table S3. The relative mRNA levels of each gene were normalized to the mRNA of *GmEIF1B and Gmactin*. Data are presented as the mean ± SE (n = 3). **b** Effect of Fe deficiency and NaHS supply on the contents of citric acid and malic acid in leaves and roots of *Glycine max* plants. Error bars represents the mean ± SE. Columns labelled with different letters indicate significant differences at *P* < 0.05. A: –Fe, 1 μM Fe; B: –Fe + NaHS, seedlings treated with 100 μM NaHS and 1 μM Fe; C: +Fe, 50 μM Fe; D: +Fe + NaHS, seedlings treated with 100 μM NaHS and 50 μM Fe

The expression abundances of organic acid metabolism-related genes were modulated by NaHS in the roots of –Fe plants (Fig. 8a). For instance, the expression levels of 2 *GmCS* genes were affected by NaHS under –Fe and + Fe conditions, and the qRT-PCR data showed that NaHS up-regulated the gene expression level of *GmCS* under –Fe conditions, but no significant effect was found under +Fe conditions. Additionally, the expression levels of the *GmACO*, *GmOGH* and *GmSDH* genes were obviously up-regulated by NaHS under –Fe and + Fe conditions. Similarly, NaHS treatment also significantly increased the gene expression levels of *GmMS* under –Fe and + Fe conditions in the roots of soybean seedlings. Moreover, the expression abundances of 2 *GmMDHs* genes were changed by NaHS, and the qRT-PCR data indicated that NaHS slightly down-regulated the expression levels of these genes under –Fe conditions, but the expression levels of these genes were significantly up-regulated under +Fe conditions. In addition, we also analyzed the citric acid and malic acid contents in the leaves and roots of soybean seedlings under –Fe and + Fe conditions. As shown in Fig. 8b, under –Fe and + Fe conditions, the citric acid contents in leaves were significantly increased by NaHS, but in the roots, NaHS treatment slightly decreased the citric acid contents under –Fe conditions. However, the malic acid content obviously decreased in response to NaHS treatment in the leaves of –Fe plants.

## Discussion

### H_2_S regulates chlorophyll biosynthesis and photosynthesis in iron-deficient plants

Soybean (*Glycine max* L.) is an agronomic crop belonging to the legume family and is the plant species with the second-highest iron (Fe) content [42]. Soybean is especially very sensitive to Fe deficiency chlorosis [42]. Exposed to Fe deficiency, chlorophyll biosynthesis and photosynthesis are significantly inhibited, and negatively affecting soybean yields [42, 43]. Fe deficiency leads to chlorosis due to decreased chlorophyll biosynthesis, which in turn causes the yellowing of younger leaves and reductions in leaf area and shoot and root dry weight [44, 45]. In addition, Fe is vital for the establishment and function of symbiotic root nodules of legumes, which are involved in nitrogen fixation [46, 47]. In low Fe conditions, the legume plants may need to develop mechanisms involved in the acquisition and utilization of Fe to protect its symbiotic organs against the low availability of Fe [48]. Therefore, soybean can be used as a new model plant in understanding the Fe deficiency tolerance mechanisms. In the present study, our results showed that Fe deficiency could significantly inhibit the nodule number and decrease the nodule dry weight in soybean plants compared with Fe sufficiency, but the application of NaHS could obviously alleviate the Fe deficiency-induced decrease in nodule number and dry weight in soybean plants (Fig. S2A and B). Moreover, under +Fe conditions, NaHS could also promote the nodule number and nodule weight in soybean plants (Fig. S2A and B). These results are consistent with previous studies showing that low Fe conditions can inhibit the nodule number in legumes [46, 47]. Moreover, Fe deficiency disrupts the chloroplast ultrastructure and degrades chloroplast protein components [7, 40], further inhibiting photosynthesis and photosystem II in plants [7]. In the present study, soybean seedlings experiencing Fe deficiency for 15 d displayed severe chlorotic characteristics with interveinal yellowing and a low chlorophyll concentration (Fig. 1 and Table 1). Similarly, a typical phenotype of chlorosis in *Arabidopsis*, tomato, and maize seedlings grown in a Fe-deficient solution has been reported [10, 11, 20]. Interestingly, NaHS significantly increased the chlorophyll content in Fe-deficient soybean seedlings, which is consistent with the results of our previous study showing that H_2_S promotes chlorophyll synthesis under Fe deficiency in maize seedlings, a strategy II plant [10]. Additionally, Fe deficiency causes a decrease in chlorophyll, leading to a reduction in photosynthesis and photosystem II in plants [49]. Moreover, chlorotic leaves caused by Fe deficiency exhibit lower stomatal apertures and lower photosynthetic rates and an eventual reduction in WUE [50]. Furthermore, Fe deficiency affects photosynthetic electron transport, with concomitant reductions in carbon assimilation and biomass [51]. In the present study, the light-response curves indicated that NaHS promoted photosynthesis and that this process was closely related to Fe acquisition in Fe-deficient plants (Fig. S3A). Additionally, water use efficiency (WUE) was also obviously enhanced by NaHS (Fig. S3D). The same results were obtained in our previous study on maize seedlings grown in an Fe-deficient solution [10]. However, NaHS had different response to photosynthesis and photosystem II between –Fe and + Fe conditions in soybean seedlings. For instance, under –Fe conditions, the application of NaHS could promote photosynthesis and chlorophyll fluorescence parameters, including PSII, ETR, Fv/Fm, and Fv`/Fm`, but under +Fe conditions, this response was not obvious and even showed a decrease (Fig. S3 and S4). The possible explanation is that under –Fe conditions, H_2_S plays a positive role in protecting plants against the damage caused by Fe deficiency, but under +Fe conditions, plant can grow and develop normally and do not need other signalling molecules, such as H_2_S. The same phenomenon is also found in maize seedlings under Fe-deficient conditions [10]. Similarly, ABA or putrescine have different responses to physiological indexes between –Fe and + Fe conditions in plants [20, 52]. Taken together, the above results indicated that H_2_S-improved photosynthesis and photosystem II were possibly dependent on Fe availability in soybean seedlings.

### H_2_S promotes iron accumulation by changing of FCR enzyme activities and iron homeostasis-related genes expression in iron-deficient plants

Fe uptake and translocation are regulated by Fe homeostasis-related genes in plants [53], and Fe deficiency-induced chlorosis results in restricted Fe mobility from old to young leaves. In the present study, NaHS significantly improved Fe accumulation in soybean seedling tissues under –Fe conditions (Table 1). Moreover, the high Fe concentration did not affect the deficiencies of other micronutrients (Mn, Cu and Zn) (Fig. S5). These results are in agreement with those from previous studies showing that NO and ABA regulate Fe accumulation and transport in tomato and Arabidopsis [11, 20]. Additionally, three consecutive activities in epidermal cell membranes are involved in the Fe uptake from the root of soybean plants [42]. The acidification of rhizosphere by the protons transported through a membrane-bound H^+^-ATPase (AHA) in the epidermal cells is the first activity. Then, the second activity is that Fe^3+^ is reduced to soluble Fe^2+^ by FCR. Finally, the iron-regulated transporter1 (IRT1) transports Fe^2+^ ions into the epidermal cells [8]. In the present study, we found that Fe deficiency-induced FCR activity in the roots of soybean seedlings during the treatments period, but NaHS inhibited the increase in Fe deficiency-induced root FCR activity (Fig. 2c), suggesting that H_2_S plays an important role in regulating the response of Fe deficiency in plants. Similarly, previous study found that FCR activity was related to the Fe concentration in soybean plants [54]. Rhizosphere acidification-mediated by proton ATPase is another important Fe-deficient response for strategy I plants, and this response has been associated with activity of a plasmalemmalocalized H^+^-ATPase. Interestingly, NaHS significantly inhibited the Fe deficiency-induced decrease in the pH of the root bathing solution. Additionally, recent evidence has shown that root apoplastic Fe can be partially remobilized under Fe deficiency [20, 55]. Here, the amount of apoplastic Fe in roots obviously decreased under Fe deficiency conditions, and this decrease was more pronounced in the presence of NaHS (Fig. 2e), indicating that H_2_S promotes the reutilization of root apoplastic Fe in soybean seedlings. The probable mechanism for this process may be as follows: H_2_S react with Fe fixed in the apoplast, making the Fe available for absorption by root cells for translocation to the shoot.

We used the transcriptome and RT-PCR methods to analyze the Fe homeostasis-related gene expression abundances, which allowed a better understanding of Fe deficiency-induced chlorosis and the role of H_2_S in Fe acquisition and photosynthesis. Our results showed that NaHS significantly stimulated the expression levels of *GmHA* genes under Fe deficiency conditions, and these results are consistent with the findings of previous studies indicating that NO and other signalling molecules can regulate the Fe-deficient response by affecting proton ATPase (*AHA*) gene expression [52, 56, 57]. In addition, previous studies have shown that the up-regulated expression of *FCR* and *IRT* might be expected to increase Fe acquisition in roots, thus strengthening plant tolerance to Fe deficiency [20, 51, 52]. Our results showed that the expression levels of *GmFCR* were obviously increased by NaHS under Fe deficiency, but two other genes, such as *GmFRO2* and *GmFRO7*, were significantly down-regulated under the same conditions (Fig. 4). One possible explanation for this phenomenon is that the H_2_S-alleviated Fe deficiency response mainly increases the expression of *GmFCR* rather than the expression abundances of *GmFRO2* and *GmFRO7* in soybean. Ferritins act as Fe storage proteins and play a major role in Fe mobilization and distribution under Fe deficiency conditions [15, 58]. Moreover, ferritins are mainly located in chloroplasts and accumulate when sufficient Fe is available in plants. Here, the expression of *Gmfer2* and *Gmfer4* were up-regulated by NaHS (Fig. 4), suggesting that H_2_S promoted Fe mobilization and distribution under Fe deficiency conditions by regulating the ferritin abundances in soybean seedlings. Similarly, previous studies have also indicated that NO and glutathione promote the accumulation of both ferritin mRNA and protein in *Arabidopsis* [58, 59]. Additionally, previous studies have suggested that bHLHs, as transcript modulators, play key roles in maintaining Fe homeostasis in plants. Here, the expression of *GmbHLH* was increased by NaHS under –Fe and + Fe conditions (Fig. 4), suggesting that H_2_S promotes the adaptability of soybean seedlings to Fe deficiency by affecting the expression abundances of transcript modulators. Soybean divalent metal transporter 1 (GmDMT1) is a soybean homologue of the NRAMP/DMT1 family of divalent metal ion transporters, and that *GmDMT1* plays an important role in nodule iron homeostasis [60]. Similarly, we found that Fe deficiency induced higher expression of *GmDMT1*, but NaHS inhibited the –Fe-induced increase in expression of this gene in the roots of soybean (Fig. 4). Taken together, these results indicated that H_2_S is a necessary signalling molecule for the expression of iron homeostasis-related genes and related enzyme activities in soybean seedlings under –Fe and + Fe conditions.

### H_2_S can regulate Sulphur-containing metabolites and Sulphur metabolism-related gene expression to cope with iron deficiency in *Glycine max* plants

Previous studies have shown that a limited S availability reduces Fe uptake and that Fe deficiency results in the modulation of sulfate uptake and assimilation [61, 62]. It is noteworthy that S supply could help plants cope with Fe shortage [61]. For instance, tomato seedlings exhibited a positive correlation between the S nutritional status of the plant and its capability of coping with Fe deficiency, which are related to the FCR activity, IRT expression and the expression of genes encoding sulfate transporter (STs) [61]. Additionally, Zuchi et al. [63] showed that in tomato plants exposed to both S and Fe starvation, no induction of FCR activity, but S deficiency seems to prevent the development of the typical responses to Fe deficiency, and Fe deficiency significantly up-regulated most of the sulfate transporter genes belonging to groups1, 2, and 4. Previous results showed this interaction between S and Fe triggers the activation of complex response mechanisms to assumingly maintain plant normal growth and development. However, unlike in previous studies, our study mainly focused on the function of H_2_S signalling rather than sulfur nutrition in improving the adaptation of soybean seedlings to Fe deficiency. For instance, under –Fe and + Fe conditions, NaHS caused a high accumulation of endogenous H_2_S, GSH and NPTs in soybean seedling leaves and roots (Table 2). These results indicated that NaHS not only directly promoted the synthesis of endogenous H_2_S but also fed into cysteine and GSH synthesis by regulating sulphur metabolism-related gene expression in roots.

The pathway of sulphur assimilation in plants is divided into two reaction sequences: sulphate reduction and cysteine synthesis [64]. Here, we found the most sulphur assimilation-related genes were regulated by NaHS under –Fe and + Fe conditions in the roots of soybean seedlings using transcriptome and qRT-PCR analysis (Fig. 5). For instance, NaHS increases the transcript levels of the *OASTL* gene and decrease *DES* gene expression in maize seedlings [10]. The same phenomenon was found in *S. oleracea* seedlings [39]. Additionally, the biosynthesis of methionine was significantly improved by NaHS under –Fe conditions by affecting the gene expression levels of *GmMetS*. Finally, we found that the transcript abundances of other S-containing compounds or enzymes, such as GR, GST, and GDH, were modulated by H_2_S under –Fe and + Fe conditions. Therefore, it was concluded that H_2_S, as a signalling molecule, could cope with Fe deficiency by increasing sulphur-containing metabolites and modulating the transcript abundances of sulphur metabolism-related genes in soybean seedlings.

### Plant hormones play an important role in H_2_S-alleviated iron deficiency in *Glycine max* plants

Plant hormones and signalling molecules are involved in the response to Fe deficiency in strategy I plants by inducing the expression of genes related to Fe acquisition and homeostasis [20, 25]. For instance, in the auxin signalling pathway, *GmAUX/IAA*, *GmGH3* and *GmSAUR* were down-regulated by NaHS under –Fe conditions, but two other genes, *GmTIR1* and *GmARF*, were significantly up-regulated under the same conditions. Moreover, our results showed that NaHS decreased the synthesis of auxin IAA in roots but increased auxin IAA in leaves under –Fe conditions (Fig. 7d), suggesting that H_2_S regulated the adaptability of Fe deficiency by affecting auxin levels and auxin-related gene expression abundances in soybean seedlings. Similarly, the AUX1-mediated auxin distribution is required for Fe-deficiency-dependent lateral root elongation [65]. Additionally, previous studies have shown that Fe deficiency leads to an increase in the synthesis of auxin and that a high concentration of auxin also enhances Fe assimilation-related gene expression [56, 66]. Moreover, our results indicated that H_2_S signalling was involved in regulating Fe assimilation by affecting the zeatin (ZA) content, a natural cytokinin (CTK) in plants, and down-regulating CTK-related gene expression abundances in soybean seedlings under –Fe conditions. A previous study has shown that CTK, as a negative regulator of Fe-deficiency responses, significantly inhibits several Fe-related genes, such as *IRT1* and *FRO1* [21], in accordance with our studies. Under –Fe conditions, NaHS enhanced the adaptability to Fe deficiency by inhibiting the synthesis of CTK and CTK-related gene expression levels in soybean seedlings (Fig. 6 and 7b). Additionally, in the gibberellin (GA) signalling pathway, the gene expression abundances of *GmGID1* and *GmDELLA* were obviously up-regulated by NaHS treatment under –Fe conditions, but TF showed a slight down-regulation under the same conditions. Moreover, the GA contents in the leaves and roots were increased by NaHS under –Fe conditions (Fig. 7c), suggesting that H_2_S signalling could positively control the GA levels by up-regulating the expression of *GmGID1* and *GmDELLA* in soybean plants under Fe deficiency conditions. Interestingly, exogenously applied GAs induced the expression of several Fe uptake-related genes under Fe-deficient and Fe-sufficient conditions in *Arabidopsis* [67]. Furthermore, a previous study has shown that GA signalling controls Fe content by regulating the expression abundances of *DELLAs* and *FIT*-related transcription factor genes [68]. Similarly, the gene expression abundances involved in the ABA signalling pathway were decreased by NaHS under –Fe conditions. Moreover, ABA synthesis in leaves and roots was inhibited by NaHS under –Fe conditions (Fig. 7a), suggesting that H_2_S may modulate ABA synthesis and the expression abundances of related genes under Fe-deficient conditions. Previous studies have shown that exogenous ABA treatment alleviates the chlorosis caused by Fe deficiency by promoting Fe transport and redistribution between shoots and roots [20]. Moreover, exogenous ABA application obviously enhances Fe uptake by regulating the expression levels of *CmbHLH1* in chrysanthemum, suggesting that ABA may be positively modulated in the process of Fe absorption [69]. In strategy I plants, the transcriptional regulation of a series of Fe-acquisition genes is controlled by ethylene (ETH) [18, 19, 70]. Moreover, the ethylene response factor *AtERF4* negatively regulate the Fe-deficient response in *Arabidopsis* [70]. Interestingly, our results showed that the transcription regulation of a series of ethylene signalling pathway-related genes was modulated by H_2_S under –Fe conditions, suggesting that ethylene together with H_2_S regulated the Fe-deficient response in soybean seedlings. Additionally, the expression abundances of brassinosteroid (BR) signalling pathway-related genes were influenced by NaHS under –Fe conditions. BRs modulate numerous physiological processes in plants [71]. They play a vital role in regulating the responses to Fe deficiency, and BR biosynthesis-defective mutants display a greater tolerance than wild type, suggesting that BRs act as negative regulators of Fe deficiency responses [72]. Jasmonates (JAs) are involved in regulating Fe deficiency in plants [23, 73]. JAs signalling are activated in the very early stages of the Fe-deficient response in rice roots, which is partly regulated by the transcription factor *IDEF1* and the ubiquitin ligases *OsHRZs* [73]. The transcript abundances of many JA signalling-related genes, including *OsJAZs*, *OsCOIs*, and *OsJARs*, are influenced by Fe deficiency [73]. In the present study, we found that these genes, including *GmJAR1*, *GmCOI1*, *GmJAZ*, and *GmMYC2*, involved in the JA signalling pathway were down-regulated by NaHS under –Fe conditions, but the JA content in roots was not affected by NaHS under –Fe conditions (Fig. 7g). In plants, the transcript abundances of *bHLH38* and *bHLH39* are dramatically induced after the application of salicylic acid (SA), which indicates a close relationship between SA and the up-regulation of the Fe-deficient responses [25, 74]. Similarly, our results showed that the transcript levels of *GmTGA* and *GmPR-1* involved in the SA signalling pathway were down-regulated by NaHS under –Fe conditions in roots. Moreover, the free SA and bound SA contents were changed by NaHS under –Fe conditions (Fig. 7e and f). Taken together, the Fe deficiency response was regulated by H_2_S signalling and a series of complex interactions among several different plant hormones by controlling biosynthesis and signal transduction-related gene expression abundances of plant hormones in soybean seedlings.

### Organic acid biosynthesis-related gene expression and organic acid contents are regulated by H_2_S in iron-deficient *Glycine max* plants

Citrate has been considered to be the most likely major candidate for Fe transport among carboxylates present in xylem sap [75]. Moreover, increased carboxylates concentrations, including citrate and malate, as well as enhanced expression of TCA cycle-related transcripts in the root tissues of plants, are important in alleviating Fe-deficient responses [76]. In addition, an increase in the TCA related enzymes such as citrate synthase (CS), malate dehydrogenase (MDH) and isocitrate dehydrogenase (NADP^+^-IDH) has been observed in the Fe deficiency several species [77]. However, until recently, there has been little information in the literature concerning the effect of H_2_S signalling on organic acid metabolism. For instance, our previous study showed that H_2_S effects Al-induced citrate secretion from barley root apices [28], suggesting that H_2_S is responsible for changes in organic acid pools under certain stress conditions. In this study, our results clearly showed that, under Fe deficiency, NaHS influenced organic acid metabolism (citric acid and malic acid) and TCA cycles-related genes transcript levels including *GmCS*, *GmACO*, *GmMDH*, *GmMS* in soybean roots (Fig. 8). Similar results were also found in a study of silicon-alleviating Fe-deficient responses [76]. Above result showed that H_2_S regulated the TCA related gene expression and organic acid synthesis, and further enhancing the adaption of Fe deficiency in soybean plants. Moreover, data from transcriptomic analyses regarding the impact of H_2_S on Fe-deficient responses in soybean were scarce. An analysis in a recent study of soybean demonstrated that soybean should be used as a new model plant for understanding the Fe deficiency tolerance mechanism and found that a series of Fe acquirement-related genes are regulated by Fe deficiency [42]. Our results further demonstrate that H_2_S signalling could directly or indirectly affect Fe acquisition and transport by controlling organic acid metabolism, as well as the transcriptional activation of Fe-deficient associated genes involved in the TCA cycle in soybean seedlings.

## Conclusion

**Fig. 9:**
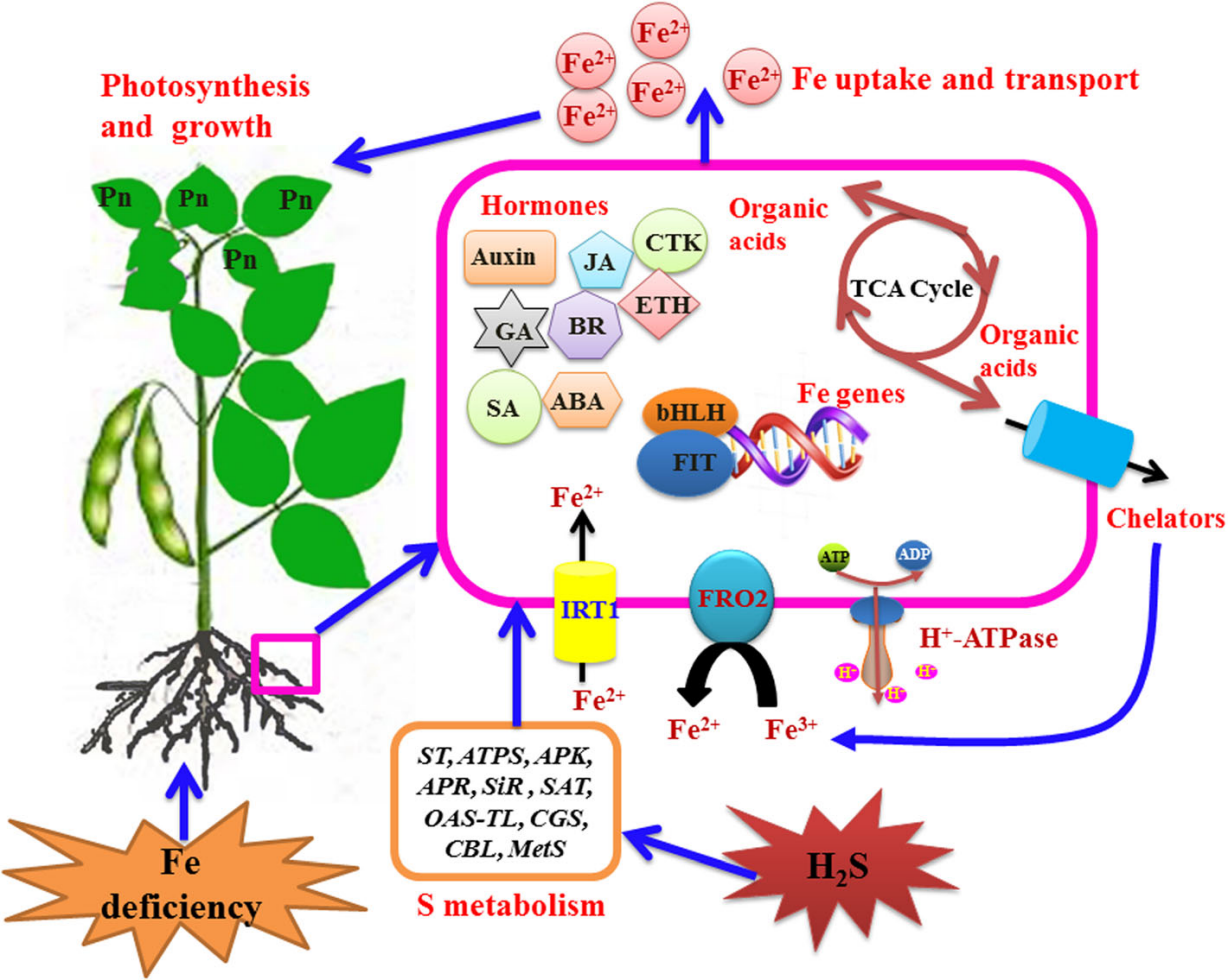
A schematic model for H_2_S-alleviated iron deficiency in roots of *Glycine max* plants. CTK, cytokinin; GA, gibberellin; ABA, abscisic acid; ETH, ethylene; BR, brassinosteroid; JA, jasmonic acid; SA, salicylic acid; IRT1, iron regulator transporter; FRO2, ferric reduction oxidase 2; bHLH, basic helix-loop-helix protein; FIT; Pn, photosynthesis; ST, sulphate transporter; ATPS, ATP sulfurylase; APK, adenylylsulphate kinase; APR, phosphoadenosine phosphosulphate reductase; SiR, sulphite reductase; SAT, serine *O*-acetyltransferase; OAS-TL, *O*-acetylserine(thiol)lyase; CGS, cystathionine gamma-synthase; CBL, cystathionine beta-lyase; MetS, methionine synthase

Based on the results shown herein and current knowledge of the mechanisms underlying plant responses to Fe deficiency, a signalling pathway by which H_2_S improves the adaptation of soybean seedlings to Fe deficiency has been proposed. As shown in Fig. 9, the present results indicated that the role of H_2_S in the alleviation of Fe deficiency chlorosis included an increase in apoplastic Fe and Fe accumulation in roots and leaves by regulating FCR activities and Fe homeostasis- and sulphur metabolism-related gene expression levels, thereby promoting plant photosynthesis and growth in soybean seedlings. In addition, exogenous H_2_S changed the concentrations of plant hormones by modulating plant hormone-related gene expression abundances in soybean seedlings grown in a Fe-deficient solution. The biosynthesis of organic acid and related gene expression also played a vital role in modulating the H_2_S-mediated alleviation of Fe deficiency in soybean seedlings.

## Methods

### Plant materials and growth conditions

Soybean seeds (*Glycine max* L. Zhonghuang 13) were obtained from Henan Academy of Agricultural Sciences. Seeds were first sterilized in 75% ethanol for 3 min, and then in a 10% sodium hypochlorite solution for an additional 10 min, followed by washing with distilled water and germinated in soil (Pindstrup Mosebrug A/S, Denmark): vermiculite (1:1) mixture for 6 d. Seedlings (six-day-old) were transferred to plastic pots (six seedlings per pot), which filled with 1.5 l of nutrient solution. The composition of the nutrient solution was as follows: 0.25 mM KH_2_PO_4_, 0.5 mM MgSO_4_, 0.5 mM Ca(NO_3_)_2_, and 1 mM KNO_3_; the composition of micronutrients was 10 μM H_3_BO_3_, 0.5 μΜ MnSO_4_, 0.5 μΜ ZnSO_4_, 0.1 μΜ CuSO_4_, and 0.1 μΜ (NH_4_)_6_Mo_7_O_24_. Iron was supplied as 50 μΜ Fe(III)-EDTA. Plants were grown under controlled environmental conditions in a growth chamber (Thermo Scientific, Thermo Fisher, USA) with a light/dark period of 14/10 h, relative humidity of 70%, temperature of 21/26 °C (night/day) and a photosynthetically active radiation (PAR) of 280 μmol m^− 2^ s^− 1^.

Seedlings were pre-cultured for 7 d in nutrient solution supplied with 50 μΜ Fe(III)-EDTA, and then grown for another 15 d in either a + Fe (50 μΜ) or –Fe (1 μΜ) nutrient solution with or without a supply of 100 μΜ NaHS, −Fe, 1 μM Fe; −Fe + NaHS, seedlings treated with 100 μM NaHS and 1 μM Fe; +Fe, 50 μM Fe; +Fe + NaHS, seedlings treated with 100 μM NaHS and 50 μM Fe. Every treatment consisted of twelve pots (three pots per replicate). Every treatment had four replicates. The pH of the nutrient solution was adjusted to 7.0 with a Delta 320 pH electrode (Mettler Toledo, Zurich, Switzerland), and it was completely renewed every 4 d and continuously aerated.

### Chlorophyll determination

The chlorophyll content was measured according to the method of Lichtenthaler [78]. Fresh soybean leaves (0.2 g) were powdered with liquid nitrogen, and four volumes of 80% (v/v) aqueous acetone were used to extract the pigment until complete bleaching was observed. The total chlorophyll content was calculated from the OD absorbance of 470, 646, and 663 nm.

### Fe content analysis

For the total Fe content analysis, the harvested roots, stems and leaves were washed three times with 1.5 mM CaCl_2,_ and then a H_2_SO_4_:H_2_O_2_ (5:1) reagent mixture was used to digest the plant samples at 420 °C in a digestion block. The total Fe concentrations were measured using an atomic absorption spectrophotometer (Hitachi Z2000, Hitachi, Japan) [10].

The apoplastic Fe content was analyzed according to Jin et al. [55]. Roots were transferred to a beaker containing 150 ml of aerated 0.5 mM CaSO_4_ for 15 min. Then the roots were put in a tube filled with 150 ml of 1.5 mM 2.2-bipyridyl. The solution was bubbled with N_2_ for 5 min before and after the addition of 12.5 mM Na_2_S_2_O_4_ to displace oxygen. Finally, the solution of the Fe(II)-bipyridyl complex was assayed spectrophotometrically at 520 nm. The blank was measured without a sample.

### Solution pH measurement

The pH of the nutrient solution was measured every 3 d with a Delta 320 pH electrode (Mettler Toledo, Zurich, Switzerland).

### Root FCR activity measurement

FCR activity was determined according to the method of Grusak [79]. Whole roots were placed in a test tube filled with 5 ml of an assay solution. The assay solution was contained 0.5 mM CaSO_4_, 0.1 mM 4-morpho-lineethanesulfonic acid, 0.1 mM bathophenanthroline-disulfonic acid disodium salt hydrate (BPDS), and 100 mM Fe(III)-EDTA and the pH of assay solution was adjusted with 1 M NaOH to 5.5. The tubes were placed in a dark room at 25 °C for 1 h, and swirled the rubes with hand for 10 min intervals. The assay solution was measured using a spectrophotometer at 535 nm, and the concentration of Fe(III) [BPDS]_3_ was calculated using an extinction coefficient of 22.14 mM^− 1^ cm^− 1^. The data are recorded as the means of relative root FCR activity.

### Determination of endogenous H_2_S, GSH, and non-protein thiols content

Endogenous H_2_S content was determined according to the method described by Sekiya et al. and Chen et al. [39, 80] with some minor modifications. The reduced GSH content was determined with a GSH reagent kit (Jiancheng Bioengineering Institute, Nanjing, China) according to the method described by Chen et al. [28]. The total content of non-protein thiols (NPTs) was analysed according to Del Longo et al. [81] with some modifications.

### RNA extraction, sequencing and library construction

Soybean root samples treated with –Fe, −Fe + NaHS, +Fe, and + Fe + NaHS for 15 d were used for RNA-Seq analysis. The transcriptome experiment was conducted with the assistance of Beijing Novogene Corporation according to the method of Wang et al. [82]. For each treatment, three individual roots were pooled together in equal amounts to generate one mixed sample, and three biological replicates were assessed for each treatment in the RNA-Seq analysis.

A total amount of 5 μg RNA per sample was used as input material for the RNA sample preparations. All samples had RIN values above 8.0. Sequencing libraries were generated using the Illumina TruSeq™ RNA Sample Preparation Kit (Illumina, San Diego, CA, USA) according to the manufacturer’s instructions. Raw image data generated from the sequencing were transformed by base calling into sequence data, which were called raw data/ raw reads and were stored in fastq format [83]. The Q20, Q30, and GC content and sequence duplication level of the clean data were simultaneously calculated. All the downstream analyses were based on high-quality clean data.

### GO and KEGG pathway

The gene ontology (GO) from the soybean genome annotation was used for the functional classification of differentially expressed genes (DEGs) according to the method of Wang et al. [82]. GO terms with corrected *P*-values < 0.05 were considered significantly enriched by DEGs.

KEGG is a database resource for understanding the high-level functions and utilities of the biological systems. The pathway is the major public pathway-related database for enrichment analysis, such as http://www.genome.jp.kegg/.

### Determination of plant hormones

Plant hormones were extracted from samples using the hormone extraction kit following the manufacturer’s instructions (Comin Biotechnology Co.Ltd., Suzhou, China, www.cominbio.com).

For ABA determination, fresh leaf and root tissues (~ 100 mg) were sealed in 2 ml tubes containing 1 ml of extraction buffer reagent I with ABA standards. The mixtures were gently agitated overnight at 4 °C and then centrifuged at 8000 g for 10 min. The upper phase was collected, and then 0.5 ml reagent II was added for decolorization by extraction three times. The water phase was adjusted to pH 2.8, combined with the organic phase in the upper layer, and then dried with a nitrogen blower. The mobile phase consisted of a constant volume of 0.5 ml, and the needle filter was filtered in the sample bottle for testing. Ten microliters of each sample was injected into a Kromasil C18 reversed-phase column (250 mm × 4.6 mm) and analysed using an HPLC system (Rigol L3000, RIGOL, Beijing, China). The mobile phase included methanol and 1% acetic acid aqueous solution. The column temperature was 30 °C and the flow rate was 0.8 ml/min. The UV detection wavelength was 254 nm, and the sampling time was 35 min. At least three independent biological replicates of each sample were performed.

For ZA and GA determination, the extraction method was the same as ABA. Ten microliters of each sample were injected into a Kromasil C18 reversed-phase column (250 mm × 4.6 mm) and analysed using an HPLC system (Rigol L3000, RIGOL, Beijing, China). The mobile phase included methanol and 1% acetic acid aqueous solution. The column temperature was 30 °C, the flow rate was 1 ml/min, the UV detection wavelength was 254 nm, and the sampling time was 40 min. At least three independent biological replicates of each sample were performed.

For IAA determination, the extraction method was the same as for ABA. Ten microliters of each sample was injected into a Kromasil C18 reversed-phase column (250 mm × 4.6 mm) and analysed using an HPLC system (Rigol L3000, RIGOL, Beijing, China). The mobile phase included methanol and ultra-pure water (400 ml:600 ml). The column temperature was 30 °C, the flow rate was 0.8 ml/min, and the sampling time was 40 min. The excitation wavelength was 275 nm and the emission wavelength was 345 nm. At least three independent biological replicates of each sample were performed.

For JA determination, the extraction method was the same as for ABA. Ten microliters of each sample were injected into a Kromasil C18 reversed-phase column (250 mm × 4.6 mm) and analysed using an HPLC system (Rigol L3000, RIGOL, Beijing, China). The mobile phase included methanol and 0.1% aqueous formic acid. The column temperature was 35 °C, the flow rate was 0.8 ml/min, and the sampling time was 40 min. The UV wavelength was 230 nm. At least three independent biological replicates of each sample were performed.

For SA determination, fresh leaf and root tissues (~ 100 mg) were ground with a mortar and added to 1 ml of precooled reagent I and extracted overnight at 4 °C. After centrifugation 8000 g for 10 min, the supernatant was collected and then the residue extracted with 0.5 ml reagent for 2 h. The supernatant was removed and combined three times, and then it was evaporated to no organic phase at 40 °C and added 20 μl reagent II with shaking for 1 min. Reagent III (1 ml) was added three times for extraction and transferred the upper organic phase in a new EP tube, dried with nitrogen, and supplemented with 0.5 ml of the mobile phase for dissolution. The samples were filtered with a needle filter. The test result was free SA. Then, 0.5 ml of reagent IV was added to the lower water phase, with shaking for 1 h, followed by the addition of 1 ml reagent III and 0.5 ml of mobile phase for dissolution. The remaining result was bound SA. Ten microliters of each sample was injected into a Kromasil C18 reverse-phase column (250 mm × 4.6 mm) and analysed using an HPLC system (Rigol L3000, RIGOL, Beijing, China). The mobile phase included 1% acetic acid aqueous solution and methanol (40:60). The column temperature was 35 °C, the flow rate was 0.8 ml/min, and the sampling time was 40 min. The excitation wavelength was 294 nm, and the emission wavelength was 426 nm. At least three independent biological replicates of each sample were performed.

### Determination of citric acid and malic acid contents

The fresh leaf and root were used to measure the citric acid and malic acid contents [76]. The samples (~ 100 mg) were immediately frozen in liquid N_2_, and ground thoroughly and extracted with 1 ml of a methanol: deionized H_2_O (3:1; v/v) mixture. All the samples were filtered with 0.22 μm-pore-size nylon syringe filters (Phenomenex, Torrance, CA, USA) before the analyses. The extract was mixed with methanol (3:1; v/v) and analyzed using an HPLC system (Agilent 1100, Agilent, SC, USA) containing a Kromasil C18 reversed-phase column (250 mm × 4.6 mm) to separate citric acid and malic acid from different samples. The column temperature, the flow rate, and the sampling time were 25 °C, 0.8 ml/min, and 20 min, respectively. The array detector was set at 214 nm.

### Validation of the mRNA-Seq data using qRT-PCR

To confirm of the RNA-Seq data, some different key genes was performed using qRT-PCR. Total RNAs were reverse-transcribed into first-strand cDNAs with M-MLV reverse transcriptase (TaKaRa, Dalian, China) according to the method of Chen et al. (2011). The 20 μl qRT-PCR reaction mixture contained the following: 1 μl of forward and reverse primers, including iron homeostasis-related genes, sulphur metabolism-related genes, plant hormone-related genes, and organic acid synthesis-related genes (Table S3), 2 μl of cDNA, and 10 μl of Faststart Universal SYBR Green Master (ROX, Mannheim, Germany). The amplification and detection of the dsDNA synthesis of these genes were performed using the PCR conditions as described in Table S4. Each sample had three independent replicates. The relative gene expression levels were calculated using the comparative threshold cycle (Ct) method. *GmEIF1B and Gmactin* were the internal control. The mRNA transcriptional abundance value of genes is expressed as 2^-ΔΔCt^ [84]. The QuantStudio™ 6 Flex qRT-PCR System (Life Technologies, Thermos, USA) was used to perform the qRT-PCR.

### Statistical analysis

For dry weight, the pH of the root bathing solution, and chlorophyll fluorescence parameters, twenty replicates were used. For the physiological and biochemical analyses, at least three replicates were conducted. Every experiment contained three biological replicates. Statistical significance was tested using one-way or two-way ANOVA in SPSS 13.0 (SPSS Inc., Chicago, IL), and the results are expressed as the mean value ± SE. Post hoc comparisons were conducted using the Tukey test at a significance level of *P* < 0.05.

## Supplementary information

**Additional file 1. **Materials and Methods. **Figure S1.** H_2_S but not other sulphur- or sodium-containing compounds derived from NaHS contributed to increased chlorophyll concentration in iron deficiency *Glycine max* seedlings. *Glycine max* seedlings were treated with 100 μM of different sulphur compounds including NaHS, Na_2_S, Na_2_SO_4_, Na_2_SO_3_, NaHSO_4_, NaHSO_3_, and NaAC for 15 d under iron deficiency condition (−Fe, 1 μM Fe). **Figure S2.** Effect of NaHS on the nodule number and nodule dry weight of iron-deficient *Glycine max* plants. The 3-w-old *Glycine max* seedlings were treated with the nutrition solution containing 1 μM Fe(III)-EDTA or 50 μM Fe(III)-EDTA with or without 100 μM NaHS for 15 days. Error bars represents the mean ± SE. Columns labelled with different letters indicate significant differences with *P* < 0.05. **Figure S3.** Effect of NaHS on net photosynthesis (Pn) (A), stomatal conductance (Gs) (B), intrancellular CO_2_ concentration (Ci) (C), and water use efficiency (WUE) (D) of iron-deficient *Glycine max* plants. The 3-w-old *Glycine max* seedlings were treated with the nutrition solution containing 1 μM Fe(III)-EDTA or 50 μM Fe(III)-EDTA with or without 100 μM NaHS for 15 days. Error bars represents the mean ± SE. –Fe, 1 μM Fe; −Fe + NaHS, seedlings treated with 100 μM NaHS and 1 μM Fe; +Fe, 50 μM Fe; +Fe + NaHS, seedlings treated with 100 μM NaHS and 50 μM Fe. **Figure S4.** Effect of NaHS on photosystem II (PSII) (A), electronic transport ratio (ETR) (B), Fv/Fm (C), and Fv`/Fm` (D) of iron-deficient *Glycine max* plants. The 3-w-old *Glycine max* seedlings were treated with the nutrition solution containing 1 μM Fe(III)-EDTA or 50 μM Fe(III)-EDTA with or without 100 μM NaHS for 15 days. Error bars represents the mean ± SE. Columns labelled with different letters indicate significant differences with *P* < 0.05. –Fe, 1 μM Fe; −Fe + NaHS, seedlings treated with 100 μM NaHS and 1 μM Fe; +Fe, 50 μM Fe; +Fe + NaHS, seedlings treated with 100 μM NaHS and 50 μM Fe. **Figure S5.** Effect of NaHS on the Cu (A-C), Mn (D-F), and Zn (G-I) concentrations of iron-deficient *Glycine max* plants. The 3-w-old *Glycine max* seedlings were treated with the nutrition solution containing 1 μM Fe(III)-EDTA or 50 μM Fe(III)-EDTA with or without 100 μM NaHS for 15 days. Error bars represents the mean ± SE. Columns labelled with different letters indicate significant differences with *P* < 0.05. **Figure S6.** The correlation test of transcriptome sequencing data of *Glycine max* roots. There are three biological repeats per treatment. –Fe, 1 μM Fe; −Fe + NaHS, seedlings treated with 100 μM NaHS and 1 μM Fe; +Fe, 50 μM Fe; +Fe + NaHS, seedlings treated with 100 μM NaHS and 50 μM Fe. **Figure S7.** Volcano plot showing DEGs in *Glycine max* seedling treated with NaHS under iron deficiency condition. Biological significant (log_2_ fold change) is depicted on the x axis and statistical significant (log_10_) is depicted on the y axis. Statistical significance was corrected at *P* < 0.05. (A) –Fe + NaHS vs –Fe; (B) + Fe + NaHS vs + Fe; (C) –Fe vs + Fe; (D) + Fe + NaHS vs –Fe + NaHS. –Fe, 1 μM Fe; −Fe + NaHS, seedlings treated with 100 μM NaHS and 1 μM Fe; +Fe, 50 μM Fe; +Fe + NaHS, seedlings treated with 100 μM NaHS and 50 μM Fe. **Figure S8.** Significantly enriched gene ontology (GO) terms (*P* < 0.05) in the gene expression numbers in the root of *Glycine max* treated with NaHS under iron deficiency condition. (A) the up-regulated genes under –Fe + NaHS vs –Fe condition; (B) the down-regulated genes under –Fe + NaHS vs –Fe condition; (C) the up-regulated genes under +Fe + NaHS vs + Fe condition; (D) the down-regulated genes under +Fe + NaHS vs + Fe condition. GO terms belong to biological processes, molecular functions, and cellular components were shown in green, blue, and organ, respectively. **Table S1.** Summary of transcriptome sequencing data of *Glycine max* roots treated with NaHS under iron deficiency condition. –Fe, 1 μM Fe; −Fe + NaHS, seedlings treated with 100 μM NaHS and 1 μM Fe; +Fe, 50 μM Fe; +Fe + NaHS, seedlings treated with 100 μM NaHS and 50 μM Fe. **Table S2.** Statistics of genes in different expression-level interval of *Glycine max* roots treated with NaHS under iron deficiency condition. –Fe, 1 μM Fe; −Fe + NaHS, seedlings treated with 100 μM NaHS and 1 μM Fe; +Fe, 50 μM Fe; +Fe + NaHS, seedlings treated with 100 μM NaHS and 50 μM Fe. **Table S3.** Sequences of forward and reverse primers used in qRT-PCR for gene expression analysis in roots of *Glycine max* seedling under iron deficiency condition. **Table S4.** Procedures of dsDNA synthesis used in qRT-PCR for gene expression analysis in roots of *Glycine max* seedlings under iron deficiency condition. **Table S5.** Iron assimilation-related gene expression levels using transcriptome in *Glycine max* roots treated with NaHS under iron deficiency condition. –Fe, 1 μM Fe; −Fe + NaHS, seedlings treated with 100 μM NaHS and 1 μM Fe; +Fe, 50 μM Fe; +Fe + NaHS, seedlings treated with 100 μM NaHS and 50 μM Fe. **Table S6.** Sulfur assimilation-related gene expression levels using transcriptome in *Glycine max* roots treated with NaHS under iron deficiency condition. –Fe, 1 μM Fe; −Fe + NaHS, seedlings treated with 100 μM NaHS and 1 μM Fe; +Fe, 50 μM Fe; +Fe + NaHS, seedlings treated with 100 μM NaHS and 50 μM Fe. **Table S7.** Plant hormones-related gene expression levels using transcriptome in *Glycine max* roots treated with NaHS under iron deficiency condition. –Fe, 1 μM Fe; −Fe + NaHS, seedlings treated with 100 μM NaHS and 1 μM Fe; +Fe, 50 μM Fe; +Fe + NaHS, seedlings treated with 100 μM NaHS and 50 μM Fe. **Table S8.** Organic acid-related gene expression levels using transcriptome in *Glycine max* roots treated with NaHS under iron deficiency condition. –Fe, 1 μM Fe; −Fe + NaHS, seedlings treated with 100 μM NaHS and 1 μM Fe; +Fe, 50 μM Fe; +Fe + NaHS, seedlings treated with 100 μM NaHS and 50 μM Fe.

## Data Availability

Our raw Illumina sequence data were deposited in the National Center for Biotechnology Information (NCBI) and be accessed in the sequence read archive (SRA) database (http://www.ncbi.nlm.nih.gov/sra). The accession number is PRJNA655258 (https://www.ncbi.nlm.nih.gov/bioproject/PRJNA655258), which includes 12 accession items (SAMN15731791-SAMN15731802). All data generated or analysed during this study are included in this published article and the supplementary information files.

## Abbreviations

CTK: Cytokinin
GA: Gibberellin
ABA: Abscisic acid
ETH: Ethylene
BR: Brassinosteroid
JA: Jasmonic acid
SA: Salicylic acid
IRT1: Iron regulator transporter
FRO2: Ferric reduction oxidase 2
bHLH: basic helix-loop-helix protein
Pn: Photosynthesis
WUE: Water use efficiency
ST: Sulfate transporter
ATPS: ATP sulfurylase
APK: Adenylylsulphate kinase
APR: Phosphoadenosine phosphosulphate reductase
SiR: Sulphite reductase
SAT: Serine *O*-acetyltransferase
OAS-TL: *O*-acetylserine(thiol)lyase
CGS: Cystathionine gamma-synthase
CBL: Cystathionine beta-lyase
MetS: Methionine synthase
