# Supplementary material for: Hydrogen sulphide alleviates iron deficiency by promoting iron availability and plant hormone levels in Glycine max seedlings
Source: BMC Plant Biol. 2020 Aug 20;20:383. doi: 10.1186/s12870-020-02601-2 (PMC7441670; doi:10.1186/s12870-020-02601-2)
Supplement: Supplementary file 1 — Additional file 1. Materials and Methods. Figure S1. H2S but not other sulphur- or sodium-containing compounds derived from NaHS contributed to increased chlorophyll concentration in iron deficiency Glycine max seedlings. Glycine max seedlings were treated with 100 μM of different sulphur compounds including NaHS, Na2S, Na2SO4, Na2SO3, NaHSO4, NaHSO3, and NaAC for 15 d under iron deficiency condition (−Fe, 1 μM Fe). Figure S2. Effect of NaHS on the nodule number and nodule dry weight of iron-deficient Glycine max plants. The 3-w-old Glycine max seedlings were treated with the nutrition solution containing 1 μM Fe(III)-EDTA or 50 μM Fe(III)-EDTA with or without 100 μM NaHS for 15 days. Error bars represents the mean ± SE. Columns labelled with different letters indicate significant differences with P < 0.05. Figure S3. Effect of NaHS on net photosynthesis (Pn) (A), stomatal conductance (Gs) (B), intrancellular CO2 concentration (Ci) (C), and water use efficiency (WUE) (D) of iron-deficient Glycine max plants. The 3-w-old Glycine max seedlings were treated with the nutrition solution containing 1 μM Fe(III)-EDTA or 50 μM Fe(III)-EDTA with or without 100 μM NaHS for 15 days. Error bars represents the mean ± SE. –Fe, 1 μM Fe; −Fe + NaHS, seedlings treated with 100 μM NaHS and 1 μM Fe; +Fe, 50 μM Fe; +Fe + NaHS, seedlings treated with 100 μM NaHS and 50 μM Fe. Figure S4. Effect of NaHS on photosystem II (PSII) (A), electronic transport ratio (ETR) (B), Fv/Fm (C), and Fv`/Fm` (D) of iron-deficient Glycine max plants. The 3-w-old Glycine max seedlings were treated with the nutrition solution containing 1 μM Fe(III)-EDTA or 50 μM Fe(III)-EDTA with or without 100 μM NaHS for 15 days. Error bars represents the mean ± SE. Columns labelled with different letters indicate significant differences with P < 0.05. –Fe, 1 μM Fe; −Fe + NaHS, seedlings treated with 100 μM NaHS and 1 μM Fe; +Fe, 50 μM Fe; +Fe + NaHS, seedlings treated with 100 μM NaHS and 50 μM Fe. Figure S5. Effect of [file 12870_2020_2601_MOESM1_ESM.docx]

**Title:** **Hydrogen sulphide alleviates iron deficiency by promoting iron availability and plant hormones levels in *Glycine max* seedlings**

**Running title: Hydrogen sulphide alleviates iron deficiency**

**Authors:** Juan Chen^1,2*^, Ni-Na Zhang^1^, Qing Pan^1^, Xue-Yuan Lin^1^, Zhouping Shangguan^1^, Jian-Hua Zhang^2^,^3^ and Ge-Hong Wei^1*^

*^1^* *State Key Laboratory of Soil Erosion and Dryland Farming on the Loess Plateau,* *Northwest A&F University, Yangling, Shaanxi 712100,* *P.R. China;*

*^2^* *School of Life Sciences and State Key Laboratory of Agrobiotechnology, the Chinese University of Hong Kong, Hong Kong*

*^3^Department of Biology, Hong Kong Baptist University, Hong Kong*

**Materials and Methods**

**Leaf gas exchange measurements**

The gas exchange in fully expanded leaves, including the net photosynthesis (Pn), stomatal conductance (Gs), and intracellular CO_2_ concentration (Ci), was determined using a portable photosynthesis system (Li-6400, Li-Cor, Lincoln, NE, USA) according to the method of Chen et al. (2015). The instantaneous water use efficiency (WUE) was calculated as the net photosynthesis rate divided by the transpiration rate (Tr).

**Determination of chlorophyll fluorescence parameters**

Chlorophyll fluorescence is a tool that can be used to interpret the stress tolerance of plants by evaluating the physiological status of the plant and the state of photosystem II (PSII) ([Prioul and Chartier 1977](#_ENREF_58); [Peeva and Cornic 2009](#_ENREF_56)). The chlorophyll fluorescence was determined using a Plant Efficiency Analyser (Hansatech Instruments Ltd., Norfolk, England). The ratio of variable (Fv) to maximum fluorescence (Fm) was measured in four seedlings per pot. The leaves were dark-adapted for more than 30 min prior to the measurement. The minimum fluorescence (Fo), the Fm, the variable fluorescence (Fv=Fm-Fo) and the ratio of Fv/Fm were recorded for 15 s at a 100% intensity level of the photon flux density (4000μmol m^-2^ s^-1^). Additionally, the steady-state fluorescence level reached upon induced by a saturating light pulse at the steady-state (Fm’), and the minimum fluorescence level after a 3 s period of far-red light required to oxidize the plastoquinone pool (Fo’) were measured and used to calculate other parameters as follows: the quantum yield of PSII photochemistry (PSII) was calculated as 1-(Fs’/Fm’). The electronic transport ratio (ETR) was calculated as PAR×PSII×0.85×0.5 (Krause and Weis, 1991).

**Figure S1** H_2_S but not other sulphur- or sodium-containing compounds derived from NaHS contributed to increased chlorophyll concentration in iron deficiency *Glycine max* seedlings. *Glycine max* seedlings were treated with 100 μM of different sulphur compounds including NaHS, Na_2_S, Na_2_SO_4_, Na_2_SO_3_, NaHSO_4_, NaHSO_3_, and NaAC for 15 d under iron deficiency condition (-Fe, 1 μM Fe).


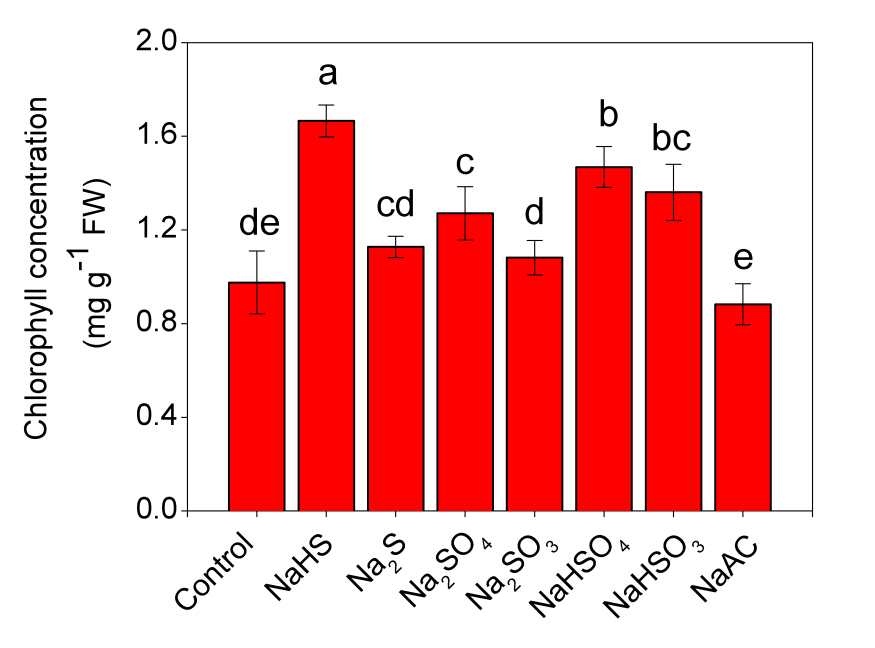


**Figure S2** Effect of NaHS on the nodule number and nodule dry weight of iron-deficient Glycine max plants. The 3-w-old *Glycine max* seedlings were treated with the nutrition solution containing 1 μM Fe(III)-EDTA or 50 μM Fe(III)-EDTA with or without 100 μM NaHS for 15 days. Error bars represents the mean ± SE. Columns labelled with different letters indicate significant differences with *P*<0.05.


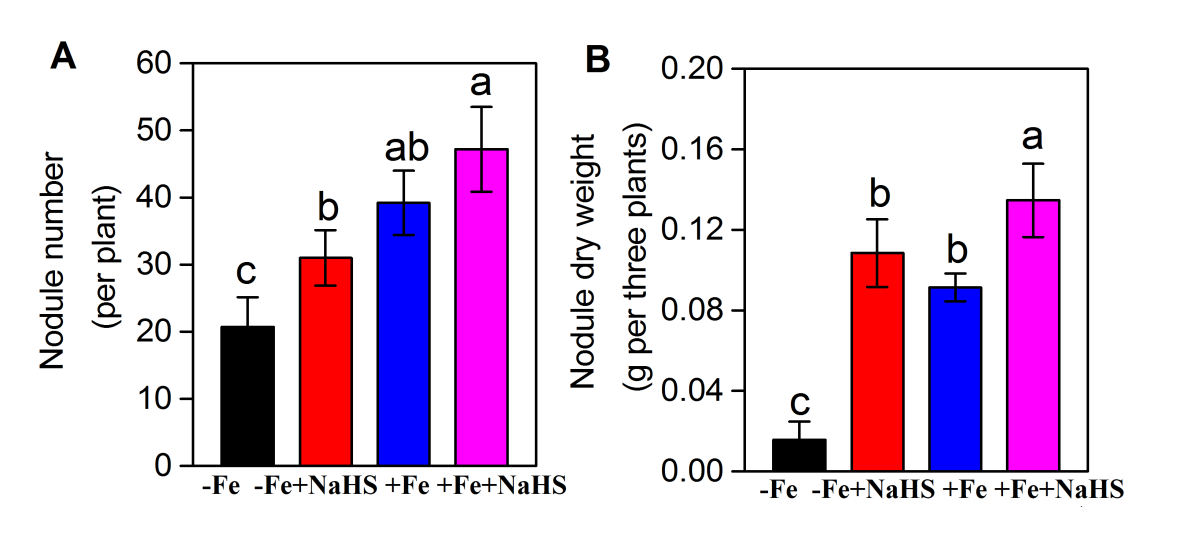


**Figure S3** Effect of NaHS on net photosynthesis (Pn) (A), stomatal conductance (Gs) (B), intrancellular CO_2_ concentration (Ci) (C), and water use efficiency (WUE) (D) of iron-deficient *Glycine max* plants. The 3-w-old *Glycine max* seedlings were treated with the nutrition solution containing 1 μM Fe(III)-EDTA or 50 μM Fe(III)-EDTA with or without 100 μM NaHS for 15 days. Error bars represents the mean ± SE. –Fe, 1 μM Fe; –Fe+NaHS, seedlings treated with 100 μM NaHS and 1 μM Fe; +Fe, 50 μM Fe; +Fe+NaHS, seedlings treated with 100 μM NaHS and 50 μM Fe.


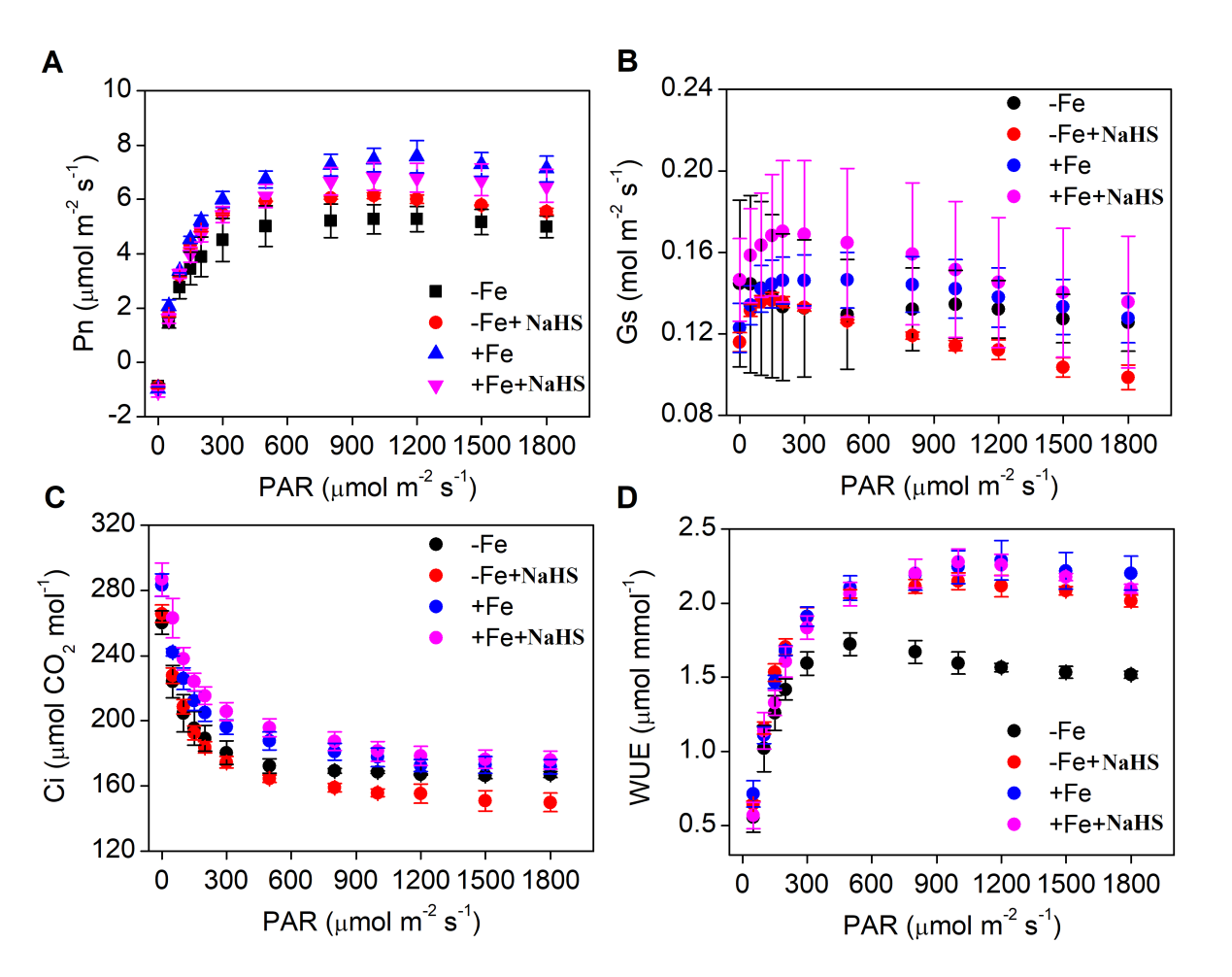


**Figure S4** Effect of NaHS on photosystem II (PSII) (A), electronic transport ratio (ETR) (B), Fv/Fm (C), and Fv`/Fm` (D) of iron-deficient *Glycine max* plants. The 3-w-old *Glycine max* seedlings were treated with the nutrition solution containing 1 μM Fe(III)-EDTA or 50 μM Fe(III)-EDTA with or without 100 μM NaHS for 15 days. Error bars represents the mean ± SE. Columns labelled with different letters indicate significant differences with *P*<0.05. –Fe, 1 μM Fe; –Fe+NaHS, seedlings treated with 100 μM NaHS and 1 μM Fe; +Fe, 50 μM Fe; +Fe+NaHS, seedlings treated with 100 μM NaHS and 50 μM Fe.


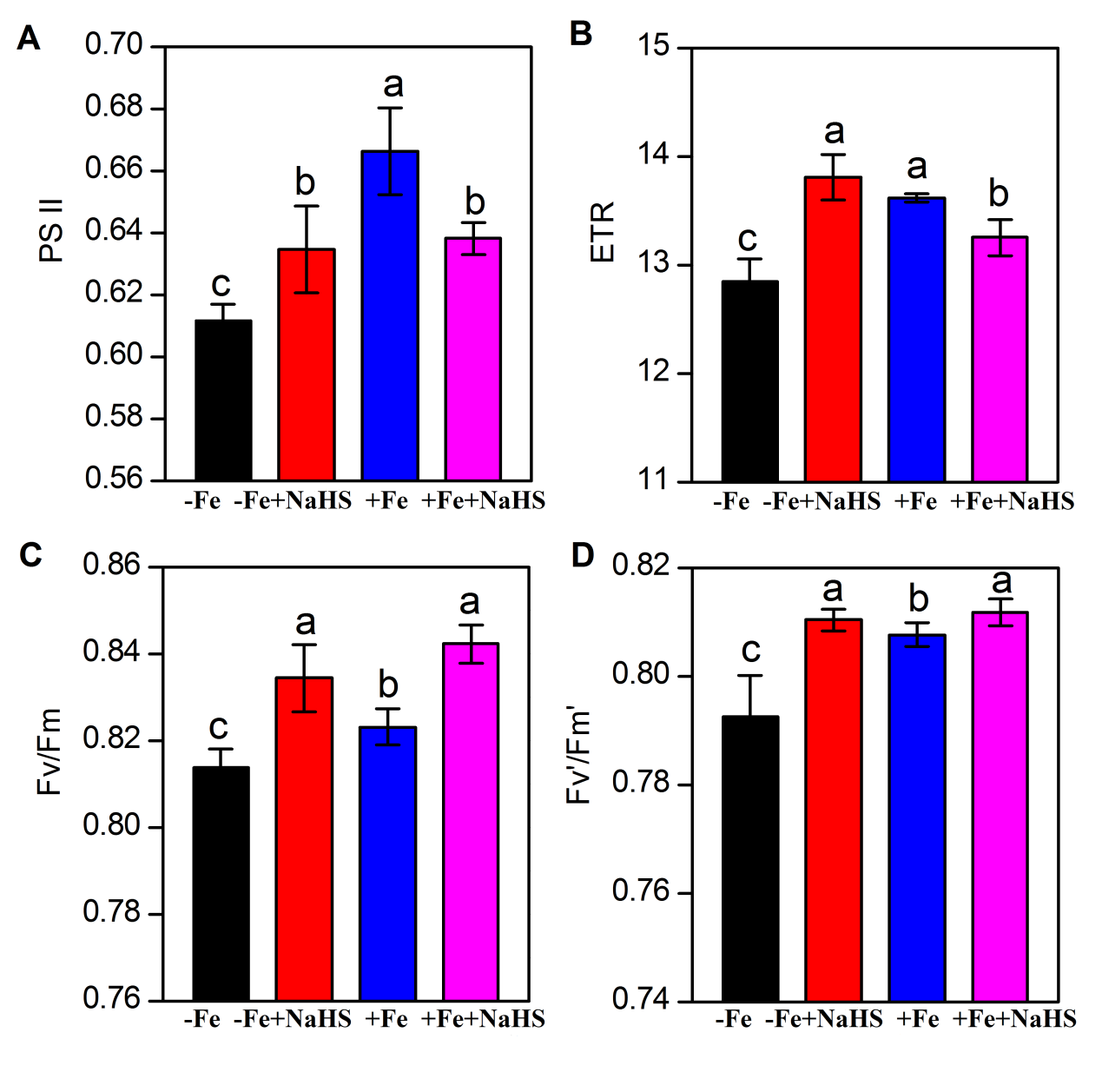


**Figure S5** Effect of NaHS on the Cu (A-C), Mn (D-F), and Zn (G-I) concentrations of iron-deficient *Glycine max* plants. The 3-w-old *Glycine max* seedlings were treated with the nutrition solution containing 1 μM Fe(III)-EDTA or 50 μM Fe(III)-EDTA with or without 100 μM NaHS for 15 days. Error bars represents the mean ± SE. Columns labelled with different letters indicate significant differences with *P*<0.05.


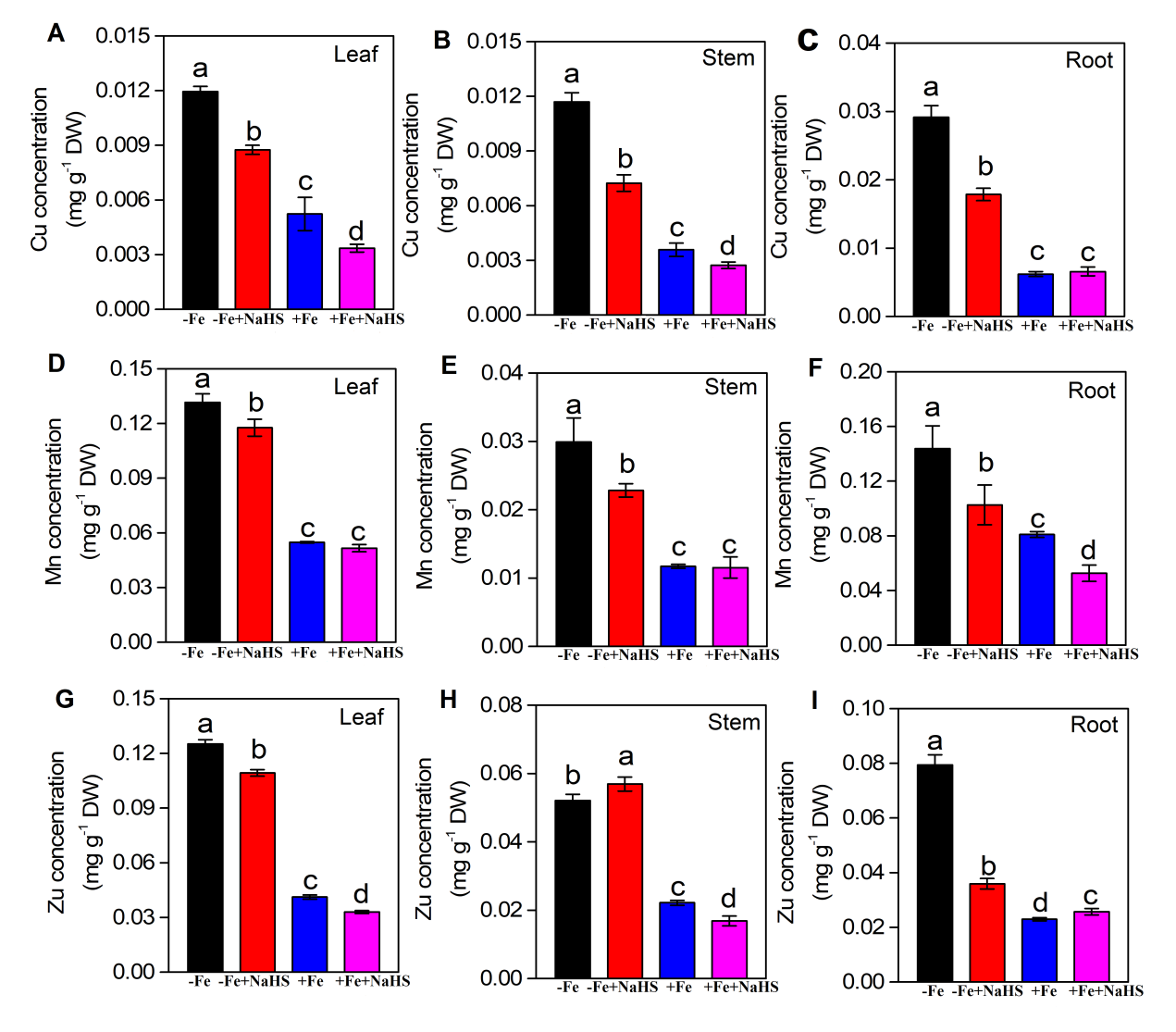


**Figure S6** The correlation test of transcriptome sequencing data of *Glycine max* roots. There are three biological repeats per treatment. –Fe, 1 μM Fe; –Fe+NaHS, seedlings treated with 100 μM NaHS and 1 μM Fe; +Fe, 50 μM Fe; +Fe+NaHS, seedlings treated with 100 μM NaHS and 50 μM Fe.


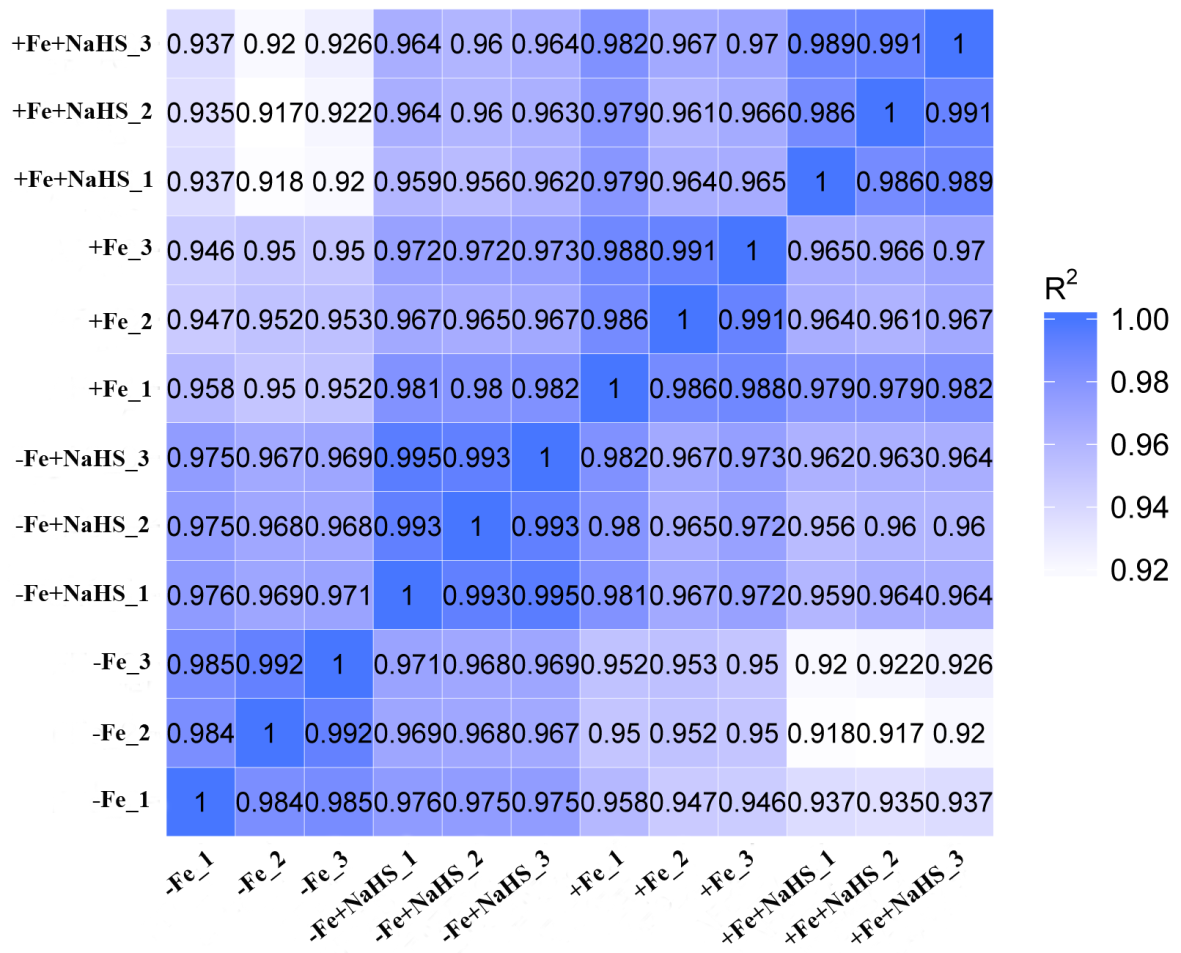


**Figure S7** Volcano plot showing DEGs in *Glycine max* seedling treated with NaHS under iron deficiency condition. Biological significant (log_2_ fold change) is depicted on the x axis and statistical significant (log_10_) is depicted on the y axis. Statistical significance was corrected at *P*<0.05. (A) –Fe+NaHS vs –Fe; (B) + Fe+NaHS vs +Fe; (C) –Fe vs +Fe; (D) +Fe+NaHS vs –Fe+NaHS. –Fe, 1 μM Fe; –Fe+NaHS, seedlings treated with 100 μM NaHS and 1 μM Fe; +Fe, 50 μM Fe; +Fe+NaHS, seedlings treated with 100 μM NaHS and 50 μM Fe.


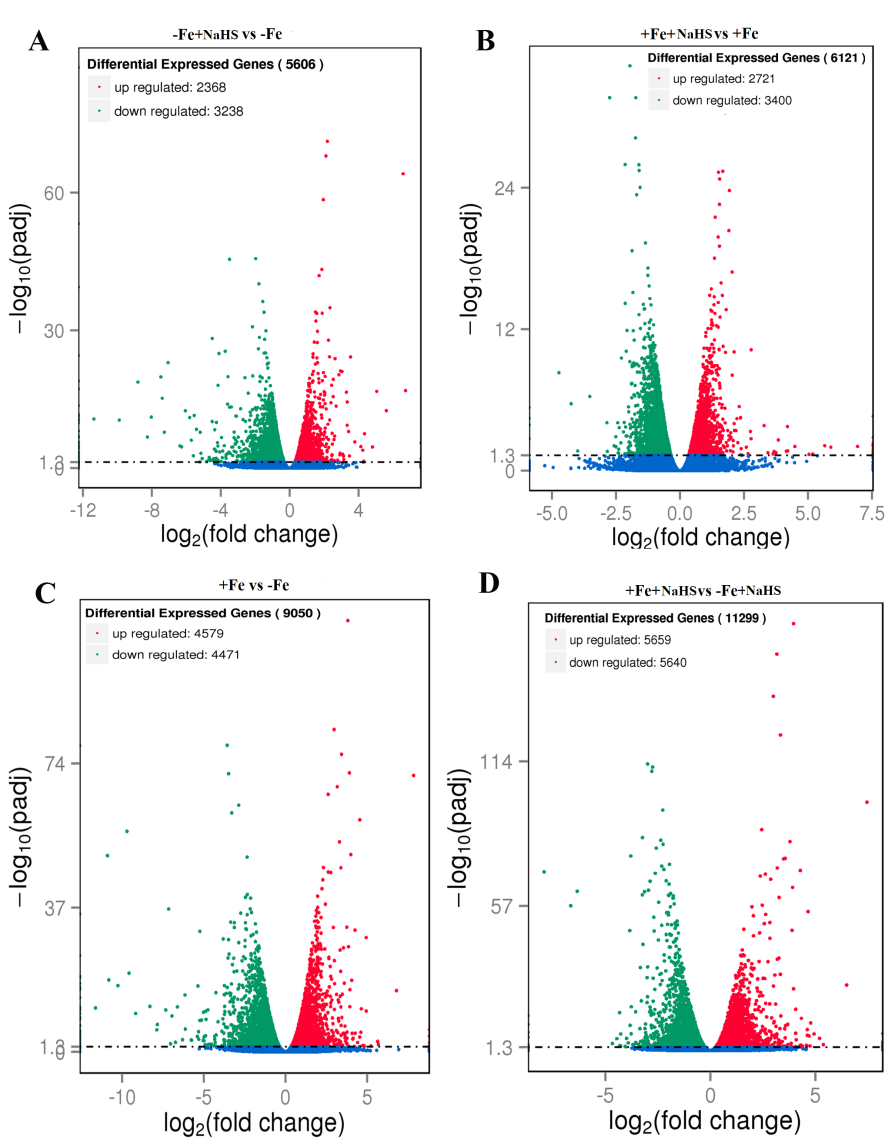


**Figure S8** Significantly enriched gene ontology (GO) terms (*P*<0.05) in the gene expression numbers in the root of *Glycine max* treated with NaHS under iron deficiency condition. (A) the up-regulated genes under –Fe+NaHS vs –Fe condition; (B) the down-regulated genes under –Fe+NaHS vs –Fe condition; (C) the up-regulated genes under +Fe+NaHS vs +Fe condition; (D) the down-regulated genes under +Fe+NaHS vs +Fe condition. GO terms belong to biological processes, molecular functions, and cellular components were shown in green, blue, and organ, respectively.


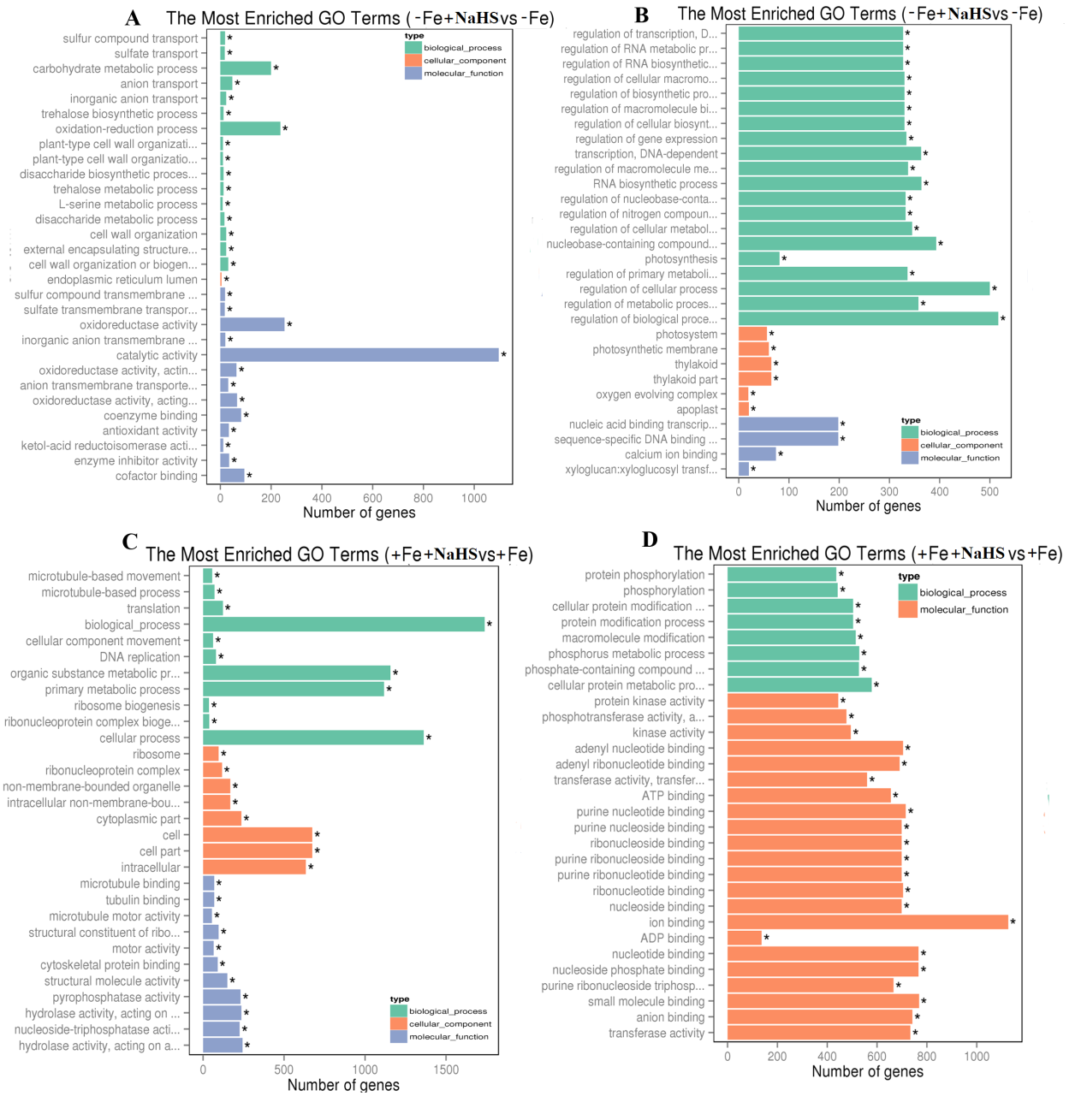


**Table S1** Summary of transcriptome sequencing data of *Glycine max* roots treated with NaHS under iron deficiency condition. –Fe, 1 μM Fe; –Fe+NaHS, seedlings treated with 100 μM NaHS and 1 μM Fe; +Fe, 50 μM Fe; +Fe+NaHS, seedlings treated with 100 μM NaHS and 50 μM Fe.

| **Sample** | **Raw Reads** | **Clean Reads (Clean/All)** | **Mapped Reads (Mapped/Clean)** | **Uniquely Mapped Reads (Unique/Clean)** |
| --- | --- | --- | --- | --- |
| -Fe_1 | 52248932 | 50315998 (96.30%) | 44874525 (89.19%) | 44223438 (87.89%) |
| -Fe_2 | 51876416 | 49545906 (95.51%) | 43560711 (87.92%) | 42903065 (86.59%) |
| -Fe_3 | 55656164 | 53647542 (96.39%) | 47481306 (88.51%) | 46787799 (87.21%) |
| -Fe+NaHS_1 | 58979796 | 56902806 (96.48%) | 49980166 (87.83%) | 49223160 (86.5%) |
| -Fe+NaHS_2 | 52603360 | 50860698 (96.69%) | 44891536 (88.26%) | 44232611 (86.97%) |
| -Fe+NaHS_3 | 66378262 | 64459732 (97.11%) | 57140857 (88.65%) | 56316897 (87.37%) |
| +Fe_1 | 62976616 | 60885868 (96.68%) | 53730445 (88.25%) | 52954652 (86.97%) |
| +Fe_2 | 68489168 | 66189828 (96.64%) | 57789311 (87.31%) | 56915362 (85.99%) |
| +Fe_3 | 53434274 | 51615554 (96.60%) | 45889410 (88.91%) | 45164446 (87.5%) |
| +Fe+NaHS_1 | 51763268 | 50140356 (96.87%) | 43902667 (87.56%) | 43293325 (86.34%) |
| +Fe+NaHS_2 | 52475554 | 50764222 (96.74%) | 44485495 (87.63%) | 43847795 (86.38%) |
| +Fe+NaHS_3 | 56203772 | 54524864 (97.01%) | 47894995 (87.84%) | 47203271 (86.57%) |

**Table S2** Statistics of genes in different expression-level interval of *Glycine max* roots treated with NaHS under iron deficiency condition. –Fe, 1 μM Fe; –Fe+NaHS, seedlings treated with 100 μM NaHS and 1 μM Fe; +Fe, 50 μM Fe; +Fe+NaHS, seedlings treated with 100 μM NaHS and 50 μM Fe.

|  |  |  | **FPKM Interval** |  |  |
| --- | --- | --- | --- | --- | --- |
| **Sample** | **0~1** | **1~3** | **3~15** | **15~60** | **>60** |
| -Fe_1 | 23422 (42.80%) | 7920 (14.47%) | 14942 (27.31%) | 6655 (12.16%) | 1779 (3.25%) |
| -Fe_2 | 24197 (44.22%) | 8025 (14.67%) | 14215 (25.98%) | 6466 (11.82%) | 1815 (3.32%) |
| -Fe_3 | 23769 (43.44%) | 8001 (14.62%) | 14540 (26.57%) | 6584 (12.03%) | 1824 (3.33%) |
| -Fe+NaHS_1 | 23635 (43.19%) | 7909 (14.45%) | 14734 (26.93%) | 6716 (12.27%) | 1724 (3.15%) |
| -Fe+NaHS_2 | 23852 (43.59%) | 7866 (14.38%) | 14589 (26.66%) | 6664 (12.18%) | 1747 (3.19%) |
| -Fe+NaHS_3 | 23761 (43.42%) | 7908 (14.45%) | 14594 (26.67%) | 6716 (12.27%) | 1739 (3.18%) |
| +Fe_1 | 24062 (43.97%) | 7763 (14.19%) | 14464 (26.43%) | 6660 (12.17%) | 1769 (3.23%) |
| +Fe_2 | 24367 (44.53%) | 7879 (14.40%) | 14163 (25.88%) | 6514 (11.90%) | 1795 (3.28%) |
| +Fe_3 | 24569 (44.90%) | 7806 (14.27%) | 13976 (25.54%) | 6566 (12.00%) | 1801 (3.29%) |
| +Fe+NaHS_1 | 23974 (43.81%) | 7635 (13.95%) | 14772 (27.00%) | 6570 (12.01%) | 1767 (3.23%) |
| +Fe+NaHS_2 | 23628 (43.18%) | 7708 (14.09%) | 15040 (27.49%) | 6663 (12.18%) | 1679 (3.07%) |
| +Fe+NaHS_3 | 23929 (43.73%) | 7602 (13.89%) | 14882 (27.20%) | 6582 (12.03%) | 1723 (3.15%) |

**Table S3** Sequences of forward and reverse primers used in qRT-PCR for gene expression analysis in roots of *Glycine max* seedling under iron deficiency condition.

| **Genebank accession** | **Gene name** | **Full name** | **Forward primer sequence (5' to 3')** | **Reverse primer sequence (5' to 3')** |
| --- | --- | --- | --- | --- |
| KF542819 | *GmIRT* | Iron regulated transporter | TTTCGGGTCTCGCCGCTATGTTC | TTCTTTGCCCTCAATCTTGGTCTCA |
| XM_003526404 | *GmHA* | Plasma membrane H^+^-ATPase | CTGAGTGGGAAGGCTTGGGATAA | CTTGTCATTGAAAAGGTTGGTTGTT |
| NM_001354789 | *Gmfer2* | Ferritin 2 | GGCAGAACGTAACAATGATCCTC | CATGACCCTTTCCAACTAATCTCA |
| NM_001250120 | *Gmfer4* | Ferritin 4 | TTACCATGCCATGTTTGCGTACTTC | CTTTTCCACCACGTTTGTTCTGATA |
| XM_003522187 | *GmFCR* | Ferrochelatase | GCTTATGACGTGGCTGCTTTAGAAT | CCGAAACAACCTTGGAAGACGAATG |
| XM_006598882.3 | *GmFRO2* | Ferric reduction oxidase 2 | GATGGTTAGTGGAGGGAGTGGAAT | TGTTGGACATATCATAGGGGGTGCC |
| XM_003534980.4 | *GmFRO7* | Ferric reduction oxidase 7 | AACGGTCATTCATTATGGTTCCAG | TGTGTGACCTGATTTCTTCAGCAAC |
| NM_001249798 | *GmDMT1* | Ferrous ion membrane transport protein | ACCCTTGCCCTTGTAGTTTCCTTTA | CCTGCTGCTAATAATCCAATACCCC |
| XM_003553410 | *GmbHLH* | Basic helix-loop-helix protein | TGGGCACAACCGATAAGATCGAAGA | CGGCAGCAGTGACAGGAACAATAAC |
| XM_003538469 | *GmST2.1* | Sulfate transporter 2.1 | TCTTAGGAATCACCCATTTTACGAC | TTGACAGAACAACGGATACAAGAGG |
| NM_001248739 | *GmATPS* | ATP sulfurylase | TCTTCGCCCAAACCTCCTT | CCTTCTTCAAATCCCTCTCAAAATC |
| NM_001248865 | *GmMetS* | Methionine synthase | CAAGGCTGTTGATGAATACAAGGAG | GGTAGGCTCATCAAACTGAATCCAT |
| NM_001360138 | *GmSAM-Mtase* | S-asenosyl-L-methionine-dependent methyltransferase | GGTATTGAAGAGGTTGAAGCAGAGG | TCACCAAGAAGGTCCAAAATCATAC |
| XM_003521094 | *GmCBL* | Cystathionine beta-lyase | GACAACATAACACTGCTCCAAACAT | TGACTCCACATCCATCTCTCTATTT |
| XM_006597590 | *GmDES* | Cysteine desulfurase | CCTCTCTCTTCCTCTTCTACTCATG | GAAGATGAATGGTAGTGTTACAGGG |
| XM_003551439 | *GmSAT1* | Serine *O*-acetyltransferase 1 | TTCAATTCACCCTTTCTCTCTCTCC | TCAACATCTAACCTTGCCTCCTCTT |
| NM_001250511 | *GmThr* | Thioredoxin | CATAGAGCCAGCGATTCACG | CGACTTCCTTCCCTTTCTTACAC |
| NM_001251077 | *GmGR* | Glutathione reductase | TCTCTCTCAACACTCTCTTCATCGC | CCAGCAACTTCTTCGGCAC |
| NM_001368757 | *GmCyS* | Cystathionine gamma-synthase | ACTGGGTAGGGGTATTGAGACTGAT | TTCATAACTCACTTGACGATTCTCC |
| NM_001249383 | *GmGST* | Glutathione S transferase | GATGAGGTGGTTCTGCTAGATTTCT | TCAATGTACTGAACAGCAACGAGAG |
| NM_001249475 | *GmGDH1* | Glutamate dehydrogenase 1 | TTTCTGTTGAGTCTTGACTTGCTTG | GTTGTGCTAAAGCATTCACTTCATC |
| XM_006598180 | *GmCS* | Citrate synthase | AGGCTGTACTGTGGCTTCTATTGAC | ATTGTATCCTCATAAGTTGGCTCCC |
| XM_006592483 | *GmMDH* | Malate dehydrogenase | GCATTCCCTCTTCTTAAAGGTGTTG | TAGATGGAGCAAATTCCTTCAAGAT |
| XM_006600736 | *GmMS* | Malate synthase | TCTTACTATGATGTGCCAGAGGGAG | CAGAGTTGAGAGCATTGATGACCAT |
| XM_003545405 | *GmEIF1B* | Eukaryotic elongation factor 1-beta | AGGAAGATAAGAAGGCAGCAGAG | ACAATAAACCAGGCATCTCAATA |
| XM_003547534 | *Gmactin* | Actin | TCGTATGAGCAAGGAAATTGG | TAGAGCCACCAATCCAGACAC |

**Table S4** Procedures of dsDNA synthesis used in qRT-PCR for gene expression analysis in roots of *Glycine max* seedlings under iron deficiency condition.

| **Gene name** | **Procedures of dsDNA synthesis** |
| --- | --- |
| *GmIRT* | 45 s at 95 °C and 40 cycles of 95 °C for 10 s, 50.6 °C for 45 s and 95°C for 15 s 60°C for 1 min, 95°C for 15 s |
| *GmHA* | 45 s at 95 °C and 40 cycles of 95 °C for 10 s, 46 °C for 45 s and 95°C for 15 s 60°C for 1 min, 95°C for 15 s |
| *Gmfer2* | 45 s at 95 °C and 40 cycles of 95 °C for 10 s, 55.9 °C for 45 s and 95°C for 15 s 60°C for 1 min, 95°C for 15 s |
| *Gmfer4* | 45 s at 95 °C and 40 cycles of 95 °C for 10 s, 57.7 °C for 45 s and 95°C for 15 s 60°C for 1 min, 95°C for 15 s |
| *GmFCR* | 45 s at 95 °C and 40 cycles of 95 °C for 10 s, 53.1 °C for 45 s and 95°C for 15 s 60°C for 1 min, 95°C for 15 s |
| *GmFRO2* | 45 s at 95 °C and 40 cycles of 95 °C for 10 s, 55.9 °C for 45 s and 95°C for 15 s 60°C for 1 min, 95°C for 15 s |
| *GmFRO7* | 45 s at 95 °C and 40 cycles of 95 °C for 10 s, 54.6 °C for 45 s and 95°C for 15 s 60°C for 1 min, 95°C for 15 s |
| *GmDMT1* | 45 s at 95 °C and 40 cycles of 95 °C for 10 s, 50.7 °C for 45 s and 95°C for 15 s 60°C for 1 min, 95°C for 15 s |
| *GmbHLH* | 45 s at 95 °C and 40 cycles of 95 °C for 10 s, 50.2 °C for 45 s and 95°C for 15 s 60°C for 1 min, 95°C for 15 s |
| *GmST2.1* | 45 s at 95 °C and 40 cycles of 95 °C for 10 s, 48.7 °C for 45 s and 95°C for 15 s 60°C for 1 min, 95°C for 15 s |
| *GmATPS* | 45 s at 95 °C and 40 cycles of 95 °C for 10 s, 46.6 °C for 45 s and 95°C for 15 s 60°C for 1 min, 95°C for 15 s |
| *GmMetS* | 45 s at 95 °C and 40 cycles of 95 °C for 10 s, 40.8 °C for 45 s and 95°C for 15 s 60°C for 1 min, 95°C for 15 s |
| *GmSAM-Mtase* | 45 s at 95 °C and 40 cycles of 95 °C for 10 s, 48.6 °C for 45 s and 95°C for 15 s 60°C for 1 min, 95°C for 15 s |
| *GmCBL* | 45 s at 95 °C and 40 cycles of 95 °C for 10 s, 55.9 °C for 45 s and 95°C for 15 s 60°C for 1 min, 95°C for 15 s |
| *GmDES* | 45 s at 95 °C and 40 cycles of 95 °C for 10 s, 57.7 °C for 45 s and 95°C for 15 s 60°C for 1 min, 95°C for 15 s |
| *GmSAT1* | 45 s at 95 °C and 40 cycles of 95 °C for 10 s, 53.1 °C for 45 s and 95°C for 15 s 60°C for 1 min, 95°C for 15 s |
| *GmThr* | 45 s at 95 °C and 40 cycles of 95 °C for 10 s, 55.9 °C for 45 s and 95°C for 15 s 60°C for 1 min, 95°C for 15 s |
| *GmGR* | 45 s at 95 °C and 40 cycles of 95 °C for 10 s, 54.6 °C for 45 s and 95°C for 15 s 60°C for 1 min, 95°C for 15 s |
| *GmCyS* | 45 s at 95 °C and 40 cycles of 95 °C for 10 s, 50.7 °C for 45 s and 95°C for 15 s 60°C for 1 min, 95°C for 15 s |
| *GmGST* | 45 s at 95 °C and 40 cycles of 95 °C for 10 s, 50.2 °C for 45 s and 95°C for 15 s 60°C for 1 min, 95°C for 15 s |
| *GmGDH1* | 45 s at 95 °C and 40 cycles of 95 °C for 10 s, 48.7 °C for 45 s and 95°C for 15 s 60°C for 1 min, 95°C for 15 s |
| *GmCS* | 45 s at 95 °C and 40 cycles of 95 °C for 10 s, 55.9 °C for 45 s and 95°C for 15 s 60°C for 1 min, 95°C for 15 s |
| *GmMDH* | 45 s at 95 °C and 40 cycles of 95 °C for 10 s, 57.7 °C for 45 s and 95°C for 15 s 60°C for 1 min, 95°C for 15 s |
| *GmMS* | 45 s at 95 °C and 40 cycles of 95 °C for 10 s, 53.1 °C for 45 s and 95°C for 15 s 60°C for 1 min, 95°C for 15 s |
| *GmEIF1B* | 45 s at 95 °C and 40 cycles of 95 °C for 10 s, 54 °C for 45 s and 95°C for 15 s 60°C for 1 min, 95°C for 15 s |
| *Gmactin* | 45 s at 95 °C and 40 cycles of 95 °C for 10 s, 48.6 °C for 45 s and 95°C for 15 s 60°C for 1 min, 95°C for 15 s |

**Table S5** Iron assimilation-related gene expression levels using transcriptome in *Glycine max* roots treated with NaHS under iron deficiency condition. –Fe, 1 μM Fe; –Fe+NaHS, seedlings treated with 100 μM NaHS and 1 μM Fe; +Fe, 50 μM Fe; +Fe+NaHS, seedlings treated with 100 μM NaHS and 50 μM Fe.

| **No.** | **Gene ID** | **Gene Name** | **Full Name** | **FPKM values** | | | |
| --- | --- | --- | --- | --- | --- | --- | --- |
|  |  |  |  | **-Fe** | **-Fe+NaHS** | **+Fe** | **+Fe+NaHS** |
| 1 | GLYMA10G36140 | HA | AAA+ ATPase domain | 1.935906405 | 3.84105177 | 2.510092194 | 4.838514569 |
| 2 | GLYMA11G35410 | HA | AAA+ ATPase | 2.570509313 | 3.840926611 | 4.90965821 | 7.692505215 |
| 3 | GLYMA20G31480 | HA | AAA+ ATPase domain | 1.648351937 | 3.416433872 | 2.037873326 | 4.342623564 |
| 1 | GLYMA05G23530 | bHLH | basic helix-loop-helix (bHLH) domain | 5.931276593 | 11.40308258 | 10.4905318 | 17.41252358 |
| 2 | GLYMA15G14890 | bHLH | basic helix-loop-helix (bHLH) domain | 24.0303468 | 37.23943573 | 32.67325174 | 44.83046278 |
| 1 | GLYMA07G19060 | Fer | Ferritin | 1.86852667 | 4.224531916 | 55.85840533 | 106.439883 |
| 2 | GLYMA18G43650 | Fer | Ferritin | 9.224327919 | 15.26464853 | 99.98705707 | 171.0471586 |

**Table S6** Sulfur assimilation-related gene expression levels using transcriptome in *Glycine max* roots treated with NaHS under iron deficiency condition. –Fe, 1 μM Fe; –Fe+NaHS, seedlings treated with 100 μM NaHS and 1 μM Fe; +Fe, 50 μM Fe; +Fe+NaHS, seedlings treated with 100 μM NaHS and 50 μM Fe.

| **No.** | **Gene ID** | **Gene Name** | **Full Name** | **FPKM values** | | | |
| --- | --- | --- | --- | --- | --- | --- | --- |
|  |  |  |  | **-Fe** | **-Fe+NaHS** | **+Fe** | **+Fe+NaHS** |
| 1 | GLYMA07G00840 | ST | Sulphate anion transporter | 2.208888885 | 4.47311134 | 4.438487662 | 8.134723204 |
| 2 | GLYMA08G19240 | ST | Sulphate anion transporter | 15.63858866 | 52.33173428 | 93.12080762 | 124.5022464 |
| 3 | GLYMA13G02060 | ST | Sulphate anion transporter | 0.758471136 | 1.802691991 | 1.994376081 | 4.469170227 |
| 4 | GLYMA13G02080 | ST | Sulphate anion transporter | 0.239869874 | 0.815583626 | 1.027593028 | 2.262308507 |
| 5 | GLYMA14G34221 | ST | Sulphate anion transporter | 0.922545889 | 1.857210569 | 0.812765025 | 1.626226546 |
| 6 | GLYMA19G34160 | ST | Sulphate anion transporter | 2.960073286 | 4.737044863 | 6.427622212 | 12.86928652 |
| 1 | GLYMA20G28980 | ATPS | ATP-sulfurylase | 58.50655761 | 91.32552122 | 86.82680913 | 94.56565481 |
| 2 | GLYMA10G38760 | ATPS | ATP-sulfurylase | 100.7471669 | 189.6917219 | 244.3121853 | 280.5884388 |
| 1 | GLYMA16G04530 | APK | Adenylylsulphate kinase | 2.094475946 | 3.33997667 | 4.940107341 | 5.181490096 |
| 1 | GLYMA07G39130 | APR | Phosphoadenosine phosphosulphate reductase | 5.908718125 | 24.93438582 | 26.92330331 | 13.03584674 |
| 2 | GLYMA15G11540 | APR | Phosphoadenosine phosphosulphate reductase | 63.28967746 | 199.8338332 | 240.514608 | 171.1814402 |
| 3 | GLYMA09G00670 | APR | Phosphoadenosine phosphosulphate reductase | 57.91637951 | 205.7800339 | 230.2623656 | 130.1834292 |
| 1 | GLYMA11G09890 | SiR | Sulphite reductase | 24.36023036 | 38.13578678 | 29.92151583 | 39.2461217 |
| 2 | GLYMA12G02200 | SiR | Sulphite reductase | 9.685270918 | 19.42054911 | 18.94904402 | 24.79056031 |
| 1 | GLYMA11G08931 | SAT | Serine acetyltransferase | 21.25975171 | 40.88387064 | 40.30735386 | 59.2276031 |
| 2 | GLYMA16G03080 | SAT | Serine acetyltransferase | 27.23973935 | 43.99216852 | 43.63844568 | 37.07054925 |
| 3 | GLYMA07G06480 | SAT | Serine acetyltransferase | 13.54007997 | 24.14318323 | 21.08121826 | 13.86663349 |
| 4 | GLYMA16G22630 | SAT | Serine acetyltransferase | 1.023955682 | 1.813237827 | 0.93885931 | 0.248384151 |
| 5 | GLYMA18G08910 | SAT | Serine acetyltransferase | 9.465725951 | 6.032214452 | 4.246575906 | 3.989020853 |
| 1 | GLYMA18G46920 | OAS-TL | O-acetylserine(thiol)lyase | 16.09279511 | 23.75188726 | 27.08330259 | 23.21432813 |
| 2 | GLYMA03G40490 | OAS-TL | O-acetylserine(thiol)lyase | 164.5021751 | 212.6299076 | 178.9670911 | 175.8809526 |
| 3 | GLYMA19G43150 | OAS-TL | O-acetylserine(thiol)lyase | 33.79670313 | 50.28617171 | 40.67112014 | 33.42847967 |
| 1 | GLYMA09G37020 | CGS | cystathionine gamma-synthase | 89.10777522 | 125.5049392 | 114.4563329 | 127.9399295 |
| 1 | GLYMA03G28530 | CBL | cystathionine beta-lyase | 6.130940161 | 7.271979215 | 7.364494982 | 10.76814584 |
| 1 | GLYMA20G28720 | MetS | Methionine synthase | 14.89848739 | 19.50016542 | 13.0720885 | 17.53545387 |
| 2 | GLYMA08G30150 | MetS | Methionine synthase | 3.81846147 | 6.197946805 | 4.751328699 | 5.352496922 |
| 3 | GLYMA16G04240 | MetS | Methionine synthase | 83.53442053 | 105.4860331 | 133.2219356 | 261.5124381 |
| 4 | GLYMA13G15140 | MetS | Methionine synthase | 19.96709406 | 18.97093045 | 14.70096392 | 20.69410286 |
| 5 | GLYMA19G29180 | MetS | Methionine synthase | 76.7776893 | 86.11586009 | 107.17841 | 197.9603839 |

**Table S7** Plant hormones-related gene expression levels using transcriptome in *Glycine max* roots treated with NaHS under iron deficiency condition. –Fe, 1 μM Fe; –Fe+NaHS, seedlings treated with 100 μM NaHS and 1 μM Fe; +Fe, 50 μM Fe; +Fe+NaHS, seedlings treated with 100 μM NaHS and 50 μM Fe.

| **No.** | **Gene ID** | **Gene Name** | **Full Name** | **FPKM values** | | | |
| --- | --- | --- | --- | --- | --- | --- | --- |
|  |  |  |  | **-Fe** | **-Fe+NaHS** | **+Fe** | **+Fe+NaHS** |
| **Auxin** |  |  |  |  |  |  |  |
| 1 | GLYMA10G02630 | TIR1 | Transport inhibitor response 1 | 4.342698612 | 5.953437533 | 6.216890429 | 7.768351176 |
| 1 | GLYMA08G21461 | AUX/IAA | AUX/IAA protein | 0.812304149 | 0.360091102 | 0.624306468 | 0.327788066 |
| 2 | GLYMA07G01800 | AUX/IAA | AUX/IAA protein | 0.445398189 | 0.109131463 | 0.368762046 | 0.230171124 |
| 3 | GLYMA01G02350 | AUX/IAA | AUX/IAA protein | 64.40657647 | 52.03002227 | 41.72672969 | 41.83890976 |
| 4 | GLYMA04G09550 | AUX/IAA | AUX/IAA protein | 19.97458008 | 13.1118707 | 9.767342484 | 8.784894464 |
| 5 | GLYMA13G43800 | AUX/IAA | AUX/IAA protein | 2.090901799 | 0.756227212 | 0.620586555 | 0.854976704 |
| 6 | GLYMA04G07040 | AUX/IAA | AUX/IAA protein | 2.14050303 | 0.77079045 | 1.604340939 | 0.824086609 |
| 7 | GLYMA02G16090 | AUX/IAA | AUX/IAA protein | 40.87368779 | 27.20261435 | 23.0088386 | 21.96372518 |
| 8 | GLYMA15G01550 | AUX/IAA | AUX/IAA protein | 2.935061358 | 1.656440226 | 1.967099668 | 3.170567265 |
| 9 | GLYMA10G03710 | AUX/IAA | AUX/IAA protein | 23.46776824 | 13.14290003 | 11.63676362 | 11.42906554 |
| 10 | GLYMA02G00260 | AUX/IAA | AUX/IAA protein | 32.50010195 | 16.87587442 | 24.36319394 | 22.57218368 |
| 11 | GLYMA19G43450 | AUX/IAA | AUX/IAA protein | 4.811704823 | 3.088999996 | 3.604283567 | 3.447217742 |
| 12 | GLYMA03G40760 | AUX/IAA | AUX/IAA protein | 67.89384293 | 51.07445492 | 61.34552206 | 52.85575152 |
| 1 | GLYMA07G32300 | ARF | Auxin response factor | 1.387198168 | 2.276041068 | 1.941929718 | 3.172617821 |
| 2 | GLYMA13G30750 | ARF | Auxin response factor | 2.0947795 | 3.045143513 | 2.790543075 | 4.090992411 |
| 3 | GLYMA03G17450 | ARF | Auxin response factor | 88.75266947 | 121.7717968 | 113.7735974 | 118.7827641 |
| 1 | GLYMA02G13910 | GH3 | GH3 auxin-responsive promoter | 48.31155033 | 28.45438882 | 28.30640883 | 23.92081905 |
| 2 | GLYMA11G05510 | GH3 | GH3 auxin-responsive promoter | 6.107859081 | 4.100820212 | 3.143562327 | 5.177030503 |
| 3 | GLYMA01G39780 | GH3 | GH3 auxin-responsive promoter | 3.304464704 | 2.107370714 | 2.185667476 | 2.765693891 |
| 4 | GLYMA06G40860 | GH3 | GH3 auxin-responsive promoter | 15.19583144 | 26.79784024 | 34.51641213 | 22.16956883 |
| 1 | GLYMA14G19670 | SAUR | Auxin responsive SAUR protein | 0.153654617 | 0.879155633 | 0.851210514 | 1.451525859 |
| 2 | GLYMA14G40530 | SAUR | Auxin responsive SAUR protein | 3.996878797 | 14.12762349 | 10.23505683 | 9.891598925 |
| 3 | GLYMA19G44805 | SAUR | Auxin responsive SAUR protein | 30.31292444 | 10.0177365 | 9.275899226 | 6.087274115 |
| 4 | GLYMA06G16640 | SAUR | Auxin responsive SAUR protein | 2.938440553 | 1.308408939 | 2.015077682 | 3.258615395 |
| 5 | GLYMA04G00890 | SAUR | Auxin responsive SAUR protein | 1.52996578 | 0.540830225 | 0.882428506 | 0.518677949 |
| 6 | GLYMA01G37215 | SAUR | Auxin responsive SAUR protein | 1.152006842 | 0.297019814 | 0.180977948 | 0.15660905 |
| 7 | GLYMA17G37610 | SAUR | Auxin responsive SAUR protein | 0.932615477 | 2.348578085 | 2.238717234 | 1.678721548 |
| 8 | GLYMA17G25180 | SAUR | Auxin responsive SAUR protein | 5.152579254 | 8.295066513 | 3.321935043 | 4.917137982 |
| 9 | GLYMA08G17880 | SAUR | Auxin responsive SAUR protein | 8.199536985 | 6.077596739 | 7.605451318 | 7.287047103 |
| 10 | GLYMA08G17880 | SAUR | Auxin responsive SAUR protein | 8.199536985 | 6.077596739 | 7.605451318 | 7.287047103 |
| 11 | GLYMA12G03771 | SAUR | Auxin responsive SAUR protein | 11.99251661 | 5.200829195 | 7.312994365 | 6.351145667 |
| 12 | GLYMA06G08340 | SAUR | Auxin responsive SAUR protein | 19.07793103 | 10.40158931 | 9.540271486 | 9.584604283 |
| 13 | GLYMA19G36771 | SAUR | Auxin responsive SAUR protein | 2.653099401 | 0.64704068 | 1.047364535 | 1.050964094 |
| **Cytokinine** |  |  |  |  |  |  |  |
| 1 | GLYMA05G28070 | CRE1 | Signal transduction histidine kinase | 16.64051899 | 13.20616234 | 7.601616973 | 5.362160801 |
| 1 | GLYMA13G28390 | AHP | Signal transduction histidine kinase, phosphotransfer domain | 111.0211137 | 79.84795102 | 72.62783093 | 54.44575395 |
| 2 | GLYMA07G38310 | AHP | Signal transduction histidine kinase, phosphotransfer domain | 24.82079189 | 15.43143948 | 12.68897444 | 10.0502418 |
| 3 | GLYMA15G10660 | AHP | Signal transduction histidine kinase, phosphotransfer domain | 59.52243319 | 44.30707283 | 46.18003173 | 47.12319539 |
| 1 | GLYMA15G24770 | B-ARR | Response regulator, plant B-type | 14.84952589 | 19.23687043 | 19.9538982 | 17.78195834 |
| 2 | GLYMA11G37480 | B-ARR | Response regulator, plant B-type | 3.127443138 | 4.860776378 | 4.172721101 | 4.44998142 |
| 3 | GLYMA18G01434 | B-ARR | Response regulator, plant B-type | 1.361043317 | 2.251593394 | 2.192721518 | 2.247135464 |
| 4 | GLYMA17G08380 | B-ARR | Response regulator, plant B-type | 0.96780859 | 1.838552863 | 1.90356146 | 2.981035597 |
| 5 | GLYMA17G03380 | B-ARR | Response regulator, plant B-type | 25.00294307 | 16.55157995 | 19.61753403 | 16.35834189 |
| 6 | GLYMA07G37220 | B-ARR | Response regulator, plant B-type | 27.56584567 | 22.46467817 | 21.49776261 | 18.55086597 |
| 7 | GLYMA13G22320 | B-ARR | Response regulator, plant B-type | 1.193936337 | 2.444771747 | 1.934898031 | 2.215142459 |
| 1 | GLYMA17G10170 | A-ARR | Signal transduction response regulator | 149.4964859 | 78.56172772 | 88.24878121 | 79.10038603 |
| 2 | GLYMA06G19870 | A-ARR | Signal transduction response regulator | 70.94653767 | 37.12187381 | 53.55329023 | 47.37937073 |
| 3 | GLYMA05G01730 | A-ARR | Signal transduction response regulator | 8.475581891 | 5.28650341 | 9.553207743 | 13.49679185 |
| 4 | GLYMA15G37770 | A-ARR | Signal transduction response regulator | 6.341947256 | 3.669762938 | 2.751004592 | 3.319724611 |
| 5 | GLYMA04G34820 | A-ARR | Signal transduction response regulator | 79.18802751 | 42.58278437 | 60.1955384 | 43.99495034 |
| **Gibberellin** |  |  |  |  |  |  |  |
| 1 | GLYMA20G37430 | GID1 | Alpha/Beta hydrolase fold | 11.39304089 | 15.48073035 | 14.02626734 | 13.61353948 |
| 1 | GLYMA04G21340 | DELLA | Transcriptional factor DELLA | 4.074190761 | 6.903857913 | 6.732266394 | 7.879645518 |
| 1 | GLYMA03G38391 | TF | Myc-type, basic helix-loop-helix (bHLH) domain | 0.383823842 | 0.104173879 | 0.066582568 | 0.114731239 |
| **Abscisic acid** |  |  |  |  |  |  |  |
| 1 | GLYMA11G35670 | PYR/PYL | Polyketide cyclase/dehydrase\|START-like domain | 7.17028887 | 10.58488402 | 10.00667616 | 10.19297541 |
| 2 | GLYMA01G12970 | PYR/PYL | Polyketide cyclase/dehydrase\|START-like domain | 5.281252511 | 3.783930021 | 2.846249141 | 3.03120845 |
| 3 | GLYMA18G43676 | PYR/PYL | Polyketide cyclase/dehydrase\|START-like domain | 17.14022172 | 22.81345952 | 19.32006959 | 15.16363802 |
| 1 | GLYMA18G03930 | PP2C | Protein phosphatase 2C | 31.63889865 | 23.68911333 | 18.88031686 | 16.29556982 |
| 2 | GLYMA11G34410 | PP2C | Protein phosphatase 2C | 20.20655921 | 16.14285101 | 11.51287542 | 12.04642024 |
| 1 | GLYMA08G20090 | SnRK2 | Serine/threonine-potein kinase | 7.517555423 | 5.157318877 | 4.53012584 | 5.791460648 |
| 2 | GLYMA06G16780 | SnRK2 | Serine/threonine-potein kinase | 10.62136565 | 7.885209271 | 11.24712212 | 9.415114623 |
| 1 | GLYMA07G33600 | ABF | Basic-leucine zipper domain (bZIP) | 27.13749805 | 21.04416847 | 22.6841396 | 23.24810938 |
| **Ethylene** |  |  |  |  |  |  |  |
| 1 | GLYMA20G21780 | ETR | Signal transduction histidine kinase, hybrid-type, ethylene sensor | 4.269016394 | 3.013961387 | 1.584490495 | 0.859680382 |
| 2 | GLYMA20G34420 | ETR | Signal transduction histidine kinase, hybrid-type, ethylene sensor | 9.108659109 | 4.566873736 | 2.726249835 | 3.53027321 |
| 1 | GLYMA10G17091 | CTR | Serine/threonine-protein kinase CTR1/EDR1 | 9.428797069 | 6.432089927 | 5.179155775 | 5.795676686 |
| 1 | GLYMA03G33850 | EIN2 | Ethylene-insensitive 2 | 10.88206986 | 7.498845552 | 5.941863674 | 7.003910397 |
| 1 | GLYMA04G07110 | EBF1/2 | F-box domain, cyclin-like | 21.67565538 | 17.70962133 | 17.59077866 | 13.52384294 |
| 2 | GLYMA14G14410 | EBF1/2 | F-box domain, cyclin-like | 13.24512144 | 8.136821797 | 14.52752861 | 8.804073694 |
| 3 | GLYMA17G12270 | EBF1/2 | F-box domain, cyclin-like | 14.66656999 | 8.868927943 | 5.714270013 | 4.781504793 |
| 4 | GLYMA04G20330 | EBF1/2 | F-box domain, cyclin-like | 7.74885816 | 4.439593205 | 4.613295728 | 4.449863455 |
| 1 | GLYMA13G03700 | EIN3 | Ethylene insensitive 3-like protein | 31.97959439 | 25.06822545 | 25.9545389 | 22.52628314 |
| 2 | GLYMA20G12250 | EIN3 | Ethylene insensitive 3-like protein | 46.08107793 | 37.19047929 | 45.32338611 | 38.02442827 |
| 1 | GLYMA03G31920 | ERF1/2 | AP2/ERF domain | 1.255694242 | 0.180708541 | 3.138389941 | 1.372831954 |
| **Brassinosteroid** |  |  |  |  |  |  |  |
| 1 | GLYMA05G24790 | BAK1 | Serine/threonine-protein kinase, active site | 4.750048216 | 3.454065438 | 3.09328607 | 2.789297282 |
| 2 | GLYMA05G24770 | BAK1 | Serine/threonine-protein kinase, active site | 3.074220305 | 1.916726508 | 3.662369248 | 1.805022153 |
| 1 | GLYMA06G15270 | BRI1 | Leucine-rich repeat-containing N-terminal, type 2 | 17.46375201 | 24.5689933 | 26.84237897 | 28.12855325 |
| 2 | GLYMA04G39610 | BRI1 | Leucine-rich repeat-containing N-terminal, type 2 | 14.65073816 | 20.94408674 | 24.36145554 | 26.90513643 |
| 1 | GLYMA03G01300 | BSK | Serine-threonine/tyrosine-protein kinase catalytic domain | 8.800518013 | 13.3251094 | 12.99468454 | 16.11395471 |
| 1 | GLYMA04G04050 | BKI1 | BRI1 kinase inhibitor 1-like | 1.921435747 | 1.115569276 | 1.314078046 | 1.362609759 |
| 1 | GLYMA17G07280 | TCH4 | Xyloglucan endotransglucosylase | 9.912110972 | 3.719998766 | 2.787280919 | 2.474124276 |
| 1 | GLYMA04G04460 | CYCD3 | Cyclin, C-terminal domain | 5.889970442 | 4.399266831 | 3.007661104 | 4.464743646 |
| **Jasmonic acid** |  |  |  |  |  |  |  |
| 1 | GLYMA19G44310 | JAR1 | GH3 auxin-responsive promoter | 14.8770648 | 11.07989721 | 14.83797184 | 13.0017725 |
| 2 | GLYMA03G41700 | JAR1 | GH3 auxin-responsive promoter | 18.82111226 | 10.69746902 | 15.09728984 | 11.06340979 |
| 1 | GLYMA02G42150 | COI1 | coronatine-insensitive protein 1 | 18.22638107 | 14.33557277 | 10.48416442 | 15.00180838 |
| 1 | GLYMA09G30460 | JAZ | CO/COL/TOC1, conserved site | 4.914798521 | 8.183769185 | 4.65263623 | 2.995931752 |
| 2 | GLYMA07G04630 | JAZ | CO/COL/TOC1, conserved site | 7.476713883 | 3.890841689 | 14.4828926 | 7.765651219 |
| 3 | GLYMA11G04130 | JAZ | CO/COL/TOC1, conserved site | 12.40545956 | 7.00383769 | 28.57798491 | 14.11291314 |
| 4 | GLYMA15G19840 | JAZ | CO/COL/TOC1, conserved site | 91.29527864 | 53.20415316 | 93.1957656 | 56.31976673 |
| 5 | GLYMA01G41290 | JAZ | CO/COL/TOC1, conserved site | 12.53352588 | 5.948913069 | 23.05642191 | 12.52140053 |
| 6 | GLYMA13G17180 | JAZ | CO/COL/TOC1, conserved site | 114.7088422 | 65.92203659 | 118.976979 | 67.9975563 |
| 7 | GLYMA17G05540 | JAZ | CO/COL/TOC1, conserved site | 26.29406123 | 15.27540042 | 42.88845391 | 26.08764358 |
| 8 | GLYMA09G08290 | JAZ | CO/COL/TOC1, conserved site | 124.0767673 | 75.72512839 | 117.8925797 | 73.19154266 |
| 9 | GLYMA16G01220 | JAZ | CO/COL/TOC1, conserved site | 8.814252048 | 4.832358834 | 15.57583981 | 10.23844734 |
| 1 | GLYMA09G33730 | MYC2 | Transcription factor MYC/MYB N-terminal | 71.71379127 | 52.00636476 | 56.87685281 | 47.55911795 |
| 2 | GLYMA08G36720 | MYC2 | Transcription factor MYC/MYB N-terminal | 27.90677411 | 20.39928273 | 19.29556567 | 23.4235852 |
| **Salicylic acid** |  |  |  |  |  |  |  |
| 1 | GLYMA08G14840 | TGA | Basic-leucine zipper domain\|DOG1 domain | 8.580595897 | 5.342923849 | 2.363630792 | 3.685365817 |
| 2 | GLYMA11G36010 | TGA | Basic-leucine zipper domain\|DOG1 domain | 84.97505818 | 65.92041045 | 59.6779503 | 50.12252753 |
| 3 | GLYMA01G21120 | TGA | Basic-leucine zipper domain\|DOG1 domain | 0.188395298 | 0.015060109 | 0.032593619 | 0.031292898 |
| 1 | GLYMA15G06780 | PR-1 | basic form of pathogenesis-related protein 1-like | 3.623794656 | 0.877231353 | 0.982335237 | 1.311368846 |
| 2 | GLYMA13G32510 | PR-1 | basic form of pathogenesis-related protein 1-like | 12.63983096 | 2.091667716 | 20.92799405 | 17.00938609 |
|  |  |  |  |  |  |  |  |

**Table S8** Organic acid-related gene expression levels using transcriptome in *Glycine max* roots treated with NaHS under iron deficiency condition. –Fe, 1 μM Fe; –Fe+NaHS, seedlings treated with 100 μM NaHS and 1 μM Fe; +Fe, 50 μM Fe; +Fe+NaHS, seedlings treated with 100 μM NaHS and 50 μM Fe.

| **No.** | **Gene ID** | **Gene Name** | **Full Name** | **FPKM values** | | | |
| --- | --- | --- | --- | --- | --- | --- | --- |
|  |  |  |  | **-Fe** | **-Fe+NaHS** | **+Fe** | **+Fe+NaHS** |
| 1 | GLYMA12G32000 | ACO | Aconitase/3-isopropylmalate dehydratase | 15.93131161 | 22.1146531 | 23.96349125 | 28.01135918 |
| 2 | GLYMA13G38480 | ACO | Aconitase/3-isopropylmalate dehydratase | 12.26388641 | 19.0701646 | 31.77185684 | 43.24902625 |
| 1 | GLYMA03G38810 | CS | succinyl-CoA synthetase-type | 28.30694562 | 13.10325445 | 16.24521842 | 10.91421654 |
| 2 | GLYMA08G17010 | CS | succinyl-CoA synthetase-type | 1.71794119 | 2.754663228 | 2.242919836 | 6.937985442 |
| 1 | GLYMA08G06820 | MDH | malate dehydrogenase | 2.316086145 | 1.465521587 | 1.44755775 | 1.31405338 |
| 2 | GLYMA17G10880 | MDH | malate dehydrogenase | 121.2523366 | 159.8587785 | 135.3144656 | 118.0265148 |
| 1 | GLYMA01G38200 | SDH | succinate dehydrogenase | 29.69113973 | 38.76804616 | 37.19608823 | 47.40654787 |
| 2 | GLYMA11G07250 | SDH | succinate dehydrogenase | 30.91693449 | 40.28991868 | 41.88821414 | 49.29183211 |
| 3 | GLYMA12G10590 | SDH | succinate dehydrogenase | 8.981662759 | 12.96486311 | 22.1229231 | 28.32761921 |
| 1 | GLYMA18G52430 | OGH | 2-oxoglutarate dehydrogenase | 27.88067339 | 37.44685245 | 37.29050135 | 39.49106001 |
| 2 | GLYMA02G10470 | OGH | 2-oxoglutarate dehydrogenase | 11.2622238 | 14.90389094 | 17.18586254 | 20.704535 |

**References**

Krause, G., and Weis, E. (1991). Chlorophyll fluorescence and photosynthesis: the basics. Annu. Rev. Plant Biol. 42, 313–349.

Peeva V, Cornic G (2009) Leaf photosynthesis of *Haberlea rhodopensis* before and during drought. Environ Exp Bot 65(2): 310-318

Prioul JL, Chartier P (1977) Partitioning of transfer and carboxylation components of intracellular resistance to photosynthetic CO_2_ fixation: A critical analysis of the methods used. Ann Bot 41(4): 789-800
